# Fast and flexible joint fine-mapping of multiple traits via the Sum of Single Effects model

**DOI:** 10.1038/s41588-025-02486-7

**Published:** 2026-02-03

**Authors:** Yuxin Zou, Peter Carbonetto, Dongyue Xie, Gao Wang, Matthew Stephens

**Affiliations:** 1 Department of Statistics, University of Chicago, Chicago, IL USA; 2 Regeneron Genetics Center, Regeneron Pharmaceuticals, Tarrytown, NY USA; 3 Department of Human Genetics, University of Chicago, Chicago, IL USA; 4 The Gertrude H. Sergievsky Center and Department of Neurology, Columbia University, New York City, NY USA

**Keywords:** Genome-wide association studies, Software

## Abstract

We introduce mvSuSiE, a multitrait fine-mapping method, to identify putative causal variants from genetic association data (individual-level or summary). mvSuSiE learns patterns of shared genetic effects from data, and exploits these patterns to improve power to identify causal single nucleotide polymorphisms (SNPs). Comparisons on simulated data show that mvSuSiE is competitive in speed, power and precision with existing multitrait methods, and uniformly improves over single-trait fine-mapping (Sum of Single Effects) performed separately for each trait. We applied mvSuSiE to jointly fine-map 16 blood cell traits using data from the UK Biobank. By jointly analyzing traits and modeling heterogeneous effect-sharing patterns, we identified a substantially larger number of causal SNPs (>3,000) than single-trait fine-mapping and achieved narrower credible sets. mvSuSiE also more comprehensively characterized how genetic variants affect blood cell traits; 68% of causal SNPs showed significant effects across more than one blood cell type.

## Abstract (web summary)

A multitrait fine-mapping method, mvSuSiE, improves power and resolution over single-trait methods for identifying putative causal variants from genetic association data.

## Main

Genome-wide association analyses have been performed for thousands of traits and have identified many genomic regions associated with diseases and complex traits^1–4^. Many statistical fine-mapping methods have been developed to prioritize putative causal single nucleotide polymorphisms (SNPs) for a single trait^5–16^, but much fewer methods are available to fine-map multiple traits simultaneously. A simple strategy to fine-map multiple traits is to fine-map each trait separately and then integrate the results post hoc. However, integration of results is not straightforward; for example, it is difficult to say whether signals identified in different single-trait analyses correspond to the same underlying causal SNP. Furthermore, analyzing each trait independently is inefficient in that it cannot exploit the potential for increased power of a multivariate analysis^17^. Therefore, it is desirable to fine-map the traits simultaneously—that is, to perform ‘multitrait fine-mapping’.

Although several methods have been developed for multitrait fine-mapping^18–26^ (Table 1), these methods have important practical limitations. For example, several methods are computationally impractical for more than a small number of traits, and most methods impose restrictive assumptions about how SNPs affect traits, such as that the effects of causal SNPs are uncorrelated across traits. These assumptions are easily violated in fine-mapping applications; for example, in the blood cell traits considered in this paper, some genetic effects are specific to subsets of the traits (for example, red blood cell (RBC) traits). There are also several methods developed for the problem of colocalization of two traits (for example, refs. ^27–30^), which have different analysis aims, but overlap with multitrait fine-mapping.

**Table 1:** Overview of available statistical methods for multitrait fine-mapping

Method	Upper limit on number of causal SNPs	Data accepted		CSs	Allows correlated traits	Models effect sharing	Sample runtimes		Software	Version
		Summary	Sufficient				2 traits	20 traits		
mvSuSiE	User-specified	Yes	Yes	Yes	Yes	Yes	41 s	2 min	R	9f28916
flashfm^a^ (ref. ^20^)	10	Yes	Yes	Yes	Yes	Yes	5 min	–	R	0.0.0.9000
MT-HESS^18^	No limit	No	No	No	Yes	Yes	>1 day	–	R	1.99
BayesSUR^21^	No limit	No	No	No	Yes	Yes	7 h	–	R	2.0-1
msCAVIAR^22^	User-specified	Yes	No	No	No	Yes	>1 day	–	Command-line	0.1
CAFEH^23^	User-specified	Yes	Yes	Yes	No	No	20 s	37 s	Python	1.0
PAINTOR^19^	User-specified	Yes	No	No	No	No	30 min	–	R	3.1
MFM^b^ (ref. ^24^)	User-specified	No	No	Yes	No	No	–	–	R	0.2-1
HyPrColoc^25^	1	Yes	No	Yes^c^	No	No	<1 s	<1 s	R	1.0
moloc^26^	1	Yes	No	Yes^c^	No	No	<1 s	–	R	0.1.0

Sample runtimes were obtained by running on datasets with *J* = 5,000 SNPs, *n* = 250,000 individuals (only relevant to methods that do not accept summary data), and *R* = 2 or 20 traits. When possible, the upper limit on the number of causal SNPs, *L*, was set to 10. In our tests, PAINTOR ran for a very long time when allowing three or more causal SNPs, so we set *L* = 2. (This was without the ‘MCMC’ option, because at the time of our trials, the ‘MCMC’ option produced unreasonable results.) moloc was computationally impractical with more than four traits. See the Supplementary Note for further details and explanation of the table columns.

^a^flashfm’s properties are determined by the single-trait fine-mapping method; for illustration, we used FINEMAP^10^. flashfm with FINEMAP was limited to at most five traits. (Another flashfm interface allows up to six traits.)

^b^MFM is for multiple case-control traits with a shared set of controls.

^c^Calculation of CSs is trivial when limiting to at most one causal SNP.

Here we introduce mvSuSiE, a fast and flexible method for multitrait fine-mapping. The name ‘mvSuSiE’ evokes its origins as an extension of the Sum of Single Effects (SuSiE) model^13^ to the multivariate analysis setting. In particular, mvSuSiE combines the SuSiE model with ideas from ref. ^31^ to learn, in a flexible way, the patterns of shared genetic effects among traits. mvSuSiE automatically adapts to the patterns of effect sharing in the particular traits being analyzed, making it widely applicable to fine-mapping any set of related traits. We also leverage ideas from ref. ^16^ to allow for the analysis of summary statistics generated from a genetic association study, which are often more accessible than individual-level data^32,33^. mvSuSiE is computationally practical for jointly fine-mapping many traits in ‘biobank scale’ datasets. We demonstrate its effectiveness compared with existing methods in simulations and by fine-mapping 16 blood cell traits in 248,980 UK Biobank samples.

## Results

### Methods overview

Consider fine-mapping *R* traits in a region containing *J* SNPs (or other biallelic loci). For each individual *i* = 1, …, *n*, let ${y}_{{ir}}$ denote trait *r* measured individual *i*, and let ${x}_{{ij}}$ denote the genotype of individual *i* at SNP *j*, encoded as the number of copies of the minor allele. We perform multitrait fine-mapping using the following multivariate linear regression model:

1 $${y}_{{ir}}={\mu }_{r}+\mathop{\sum }\limits_{j=1}^{J}{x}_{{ij}}{b}_{{jr}}+{e}_{{ir}}$$

where ${\mu }_{r}$ reflects the mean of trait *r*, ${b}_{{jr}}$ is the effect of SNP *j* on trait *r* and the ${e}_{{ir}}$ s are normally distributed error terms (which may be correlated among the *R* traits). Within this regression model, we frame fine-mapping as a ‘variable selection problem’—most SNPs are assumed to have no effect on any trait, that is, most effects ${b}_{{jr}}$ are zero—and the goal of multitrait fine-mapping is to identify which SNPs have a nonzero effect on which traits, and to assess uncertainty in these inferences. (For brevity, we use the term ‘causal SNP’ to mean a SNP with a nonzero effect.) Our mvSuSiE method achieves this goal by extending the SuSiE model^13^ to the multivariate setting. By extending ideas from ref. ^16^, mvSuSiE can perform fine-mapping using either individual-level data (genotypes and phenotypes) or summary data (for example, linkage disequilibrium (LD) matrix and marginal *z* scores); see Methods and Supplementary Note for details.

Among existing approaches to fine-mapping, mvSuSiE is most closely related to CAFEH^23^, which also extends SuSiE to perform multitrait fine-mapping. Both CAFEH and mvSuSiE inherit much of the simplicity and benefits of single-trait SuSiE. Like SuSiE, both mvSuSiE and CAFEH require the user to specify an upper bound, *L*, on the number of causal SNPs in a region, and are robust to this upper bound being larger than needed. And both methods exploit SuSiE’s simple fitting procedure, Iterative Bayesian Stepwise Selection (IBSS)^13^. IBSS is similar to standard forward stepwise selection but improves it by (1) using Bayesian computation to account for uncertainty in SNP selection at each step and (2) iteratively updating selection to correct errors in initial selections as fitting progresses. However, mvSuSiE also improves on CAFEH in the following two key ways:

1. mvSuSiE uses a flexible prior distribution—specifically, a mixture of multivariate normal distributions, as in ref. ^31^—to model effect-sharing patterns across traits. Furthermore, the parameters of this prior are estimated from the data, allowing mvSuSiE to adapt to each dataset. This flexible approach allows for different causal SNPs that show different patterns of association; for example, in analyses of blood cell traits (below), mvSuSiE learns that some SNPs affect RBC (erythrocyte) traits primarily, some affect primarily white blood cell (leukocyte) traits, and some affect both, or a subset of one or the other. In contrast, CAFEH assumes a less flexible and less adaptive prior in which causal effects are independent across traits.
2. mvSuSiE allows correlations among measurements of traits, with these correlations again estimated from the data. In contrast, CAFEH assumes that measurements are independent across traits, which is an inappropriate assumption in association studies involving correlated traits.

For the first key way, estimating the prior distribution from the data involves combining information across many causal SNPs from many regions, which is an additional step compared with standard single-trait fine-mapping analyses. This additional step can be avoided by using a simpler fixed prior (Supplementary Note), but at the potential loss of power.

We also introduce new methods for summarizing inferences from multitrait fine-mapping. Again, this builds on SuSiE, which summarizes single-trait results by reporting, for each SNP, a posterior inclusion probability (PIP) quantifying the probability that the SNP is causal, and by reporting ‘credible sets’ (CSs)^7,13^ that are designed to capture, with high probability, at least one causal SNP. Informally, each CS represents an independent association signal in the data, and the size of a CS (that is, the number of SNPs in the CS) indicates how precisely one can pinpoint the causal SNP underlying this signal. For multitrait analyses, it may seem natural to report PIPs and CSs separately for each trait. However, this raises thorny issues—for example, if the reported CSs for two traits overlap, do they represent the same signal from a single underlying causal SNP, or different signals from multiple causal SNPs? To avoid these problems, we separate inference into two questions.

First question is as follows: which SNPs are causal for ‘at least one trait’? This question is answered by ‘cross-trait PIPs and CSs’ that summarize the inferences across all traits.

Second question is as follows: for each causal SNP (that is, CS) identified, which traits does it affect? This is answered by computing a ‘trait-wise’ measure of significance, the ‘local false sign rate (lfsr)’^31,34^, for each SNP in each trait. (A small lfsr indicates a high confidence in the ‘sign’ of the effect.) Because SNPs in a CS are typically in high LD, their trait-wise lfsr values are typically similar, and it is convenient to use a single number, the ‘average lfsr’, as a trait-wise measure of significance of each CS. If the ‘average lfsr’ for trait *r* is small, this indicates high confidence in the sign of the effect—that is, a small posterior probability that the true effect is zero or that its estimated sign is incorrect—and we say the CS is ‘significant for trait *r’*.

**Fig. 1:**
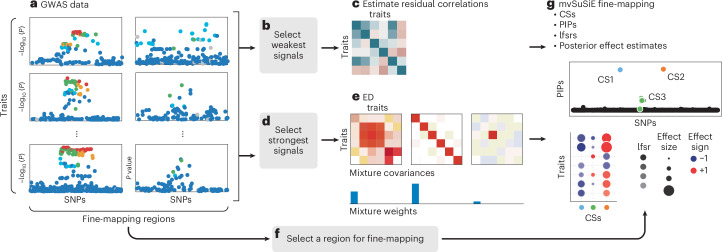
Overview of multivariate fine-mapping using mvSuSiE. mvSuSiE accepts as input *R* traits and SNP genotypes measured in *n* individuals, and *M* target fine-mapping regions. **a**, Alternatively, mvSuSiE-RSS accepts SNP-level summary statistics computed from these data. **b**,**c**, The weakest SNP association signals are extracted from these data (**b**) and used in **c** to estimate correlations in the trait residuals. **d**,**e**, Separately, the strongest association signals are extracted (**d**) to estimate effect-sharing patterns (**e**) using ED^53^. **f**,**g**, Finally, the effect-sharing patterns estimated by ED, together with the estimated weights, are used to construct a prior for the unknown multivariate effects, and this prior is used in mvSuSiE to perform multivariate fine-mapping simultaneously for all SNPs within a selected region. Steps in **f** and **g** are repeated for each fine-mapping region of interest. The key mvSuSiE outputs are: a list of CSs, each of which is intended to capture a causal SNP; a PIP for each SNP giving the probability that the SNP is causal for at least one trait; ‘average lfsr’ summarizing the significance of each CS in each trait; and SNP-wise posterior effect estimates on each trait. For example, if a region contains three causal SNPs, mvSuSiE will, ideally, output three CSs, each containing a true causal SNP, with the ‘average lfsr’ indicating which traits are significant for each CS. See Methods for definitions. ED, extreme deconvolution.

In summary, the reported results from the mvSuSiE analysis comprise the ‘cross-trait PIPs and CSs’ and ‘trait-wise’ measures of significance (lfsr) for each SNP and each CS in each trait. Figure 1 summarizes the mvSuSiE analysis workflow for a typical genetic association study.

### Evaluation in simulations using UK Biobank genotypes

We compared mvSuSiE with existing multitrait fine-mapping methods and a single-trait fine-mapping method, SuSiE^13,16^, in simulations. Among available multitrait fine-mapping methods (Table 1), MT-HESS^18^ and BayesSUR^21,35,36^ are similar to mvSuSiE in features and modeling assumptions, but are computationally impractical for large fine-mapping datasets. msCAVIAR^22^ shares mvSuSiE’s ability to model effect sharing, but is designed to analyze data from multiple studies and therefore makes modeling assumptions that are less appropriate for multiple traits. MFM^24^ is another multitrait fine-mapping method, but is specific to multiple case-control traits with a shared set of controls. Therefore, we focused our comparisons on CAFEH^23^ that can handle large multitrait fine-mapping datasets. We also compared with flashfm^20^ and PAINTOR^19^ on smaller fine-mapping datasets with two traits.

To make our simulations reflect current large-scale genomic datasets, we obtained imputed genotype data from the UK Biobank^37^ and simulated quantitative traits with one to five simulated causal SNPs per fine-mapping region. We simulated from a variety of effect-sharing patterns, with effect sizes scaled to roughly reproduce the distributions of *z* scores observed in genome-wide association analyses of complex traits from UK Biobank data. The fine-mapping regions were drawn from autosomal chromosomes and varied in size (0.4–1.6 Mb), number of SNPs (1,000–5,000 SNPs) and LD patterns.

We simulated traits under two scenarios:

1. ‘Trait-specific + shared effects’, in which SNP effects on 20 independent traits were either specific to 1 trait or shared among traits in simple ways (for example, equal effects on a pair of traits and no effect on the remaining traits)
2. ‘Complex shared effects’, in which SNP effects on 16 correlated traits were generated from a variety of sharing patterns derived from the UK Biobank blood cell traits

To compare with PAINTOR and flashfm, we also simulated smaller datasets with two independent traits and shared effects.

**Fig. 2:**
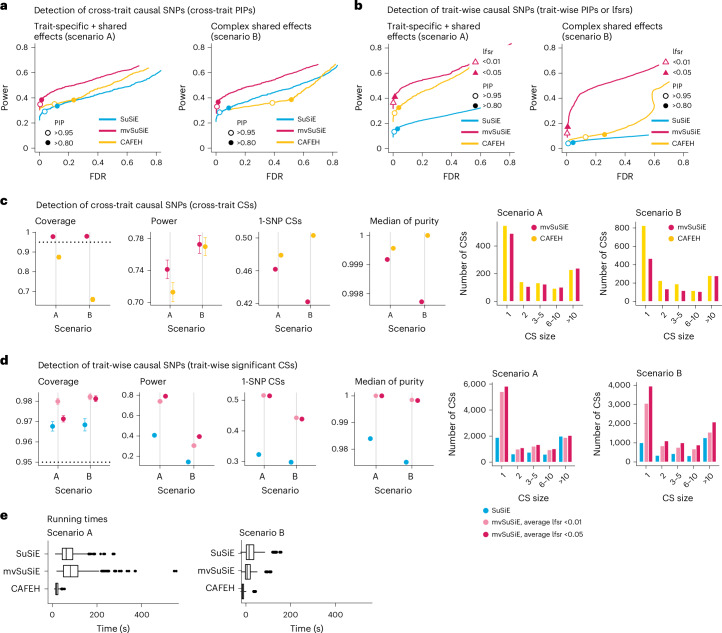
Comparison of fine-mapping methods in simulated data. **a**,**b**, Power versus FDR in identifying cross-trait (**a**) or trait-wise (**b**) causal SNPs, using SNP-wise measures. In each scenario, FDR and power were calculated by varying the measure threshold from 0 to 1 (*n* = 600 simulations). FDR = FP/(TP + FP) and power = TP/(TP + FN), where FP, TP, FN and TN denote the number of false positives, true positives, false negatives and true negatives, respectively. The specific SNP-wise measures used in **a** are PIP (mvSuSiE, CAFEH) and max-PIP (SuSiE); in **b**, PIP (SuSiE), ‘min-lfsr’ (mvSuSiE) and study PIP (CAFEH). In **a** and **b**, power and FDR at specific thresholds are indicated by the circles and triangles. Of note, the thresholds for the different methods are not equivalent or comparable; results are shown at these thresholds for illustration only (Supplementary Table 1). **c**,**d**, Evaluating the 95% cross-trait (**c**) and trait-wise (**d**) CSs from the *n* = 600 simulations using the following metrics: ‘coverage’, the proportion of CSs containing a true causal SNP; ‘power’, the proportion of true causal SNPs included in at least one CS; the proportion of CSs that contain a single SNP (‘1-SNP CSs’); and ‘median purity’, in which ‘purity’ is defined as the smallest absolute correlation (Pearson’s *r*) among all SNP pairs in a CS. Histograms of CS sizes (number of SNPs in a 95% CS) are given for each scenario. Target coverage (95%) is shown as a dotted horizontal line. Error bars show twice the empirical s.e. from the results in all simulations. **e**, Summary of runtimes (*n* = 600 simulations); the SuSiE runtimes are for running SuSiE independently on ‘all’ traits. The box plot whiskers depict 1.5× interquartile range, the box bounds represent the upper and lower quartiles (25th and 75th percentiles), the centerline represents the median (50th percentile) and points represent outliers. Of note, SuSiE analyzes each trait independently and therefore is not included in **c**. CAFEH does not provide trait-wise CSs and therefore is not included in **d**.

We compared methods for detecting cross-trait causal SNPs—in which we define a cross-trait causal SNP as one that affects at least one trait—and trait-wise causal SNPs. We assessed the performance of both SNP-wise measures (for example, PIPs) and CSs for these tasks. In practice, we recommend focusing on CS-based inferences (Fig. 2c,d) rather than SNP-wise measures (Fig. 2a,b) because the CSs account for uncertainty in the causal SNP due to LD. (In our multitrait fine-mapping of blood cell traits below, we focused on CS-based inferences.) However, not all competing methods provide comparable CS-based inferences (for example, CAFEH does not provide trait-wise CSs), so for completeness and to allow comparisons with other methods, we also evaluated the performance of SNP-wise significance measures (Fig. 2a,b).

In all our comparisons, mvSuSiE improved power, coverage and resolution (purity, proportion of 1-SNP CSs) over the SuSiE single-trait analyses (Fig. 2a,b,d; *n* = 600 simulations). The greatest gains were in scenario B, where mvSuSiE had the advantage of accounting for correlations among traits. Comparing CAFEH and single-trait SuSiE in SNP-wise inferences, CAFEH improved performance in scenario A but performed slightly less well in detecting causal SNPs in scenario B (Fig. 2a,b). CAFEH also produced poorly calibrated PIPs in scenario B (Supplementary Fig. 1 and Supplementary Table 1); for example, CAFEH at a seemingly stringent ‘study PIP’ threshold of 0.95 resulted in a false discovery rate (FDR) of 0.13 that was much higher than mvSuSiE at an lfsr threshold of 0.05 (FDR = 0.0065). This also illustrates the difficulty of setting comparable thresholds for the different quantities outputted by different methods; therefore, following the common practice in statistical fine-mapping papers, we presented results using power–FDR curves to sidestep this difficulty.

Comparing CSs (Fig. 2c,d), CAFEH improved the purity of the CSs and the proportion of 1-SNP CSs, but these improvements were tempered by CAFEH’s reduced power and coverage, particularly in scenario B. A partial explanation for these results is that scenario B contradicts CAFEH’s assumptions of independent traits and independent causal effects. In support of this explanation, when we forced mvSuSiE to make the same independence assumptions as CAFEH, mvSuSiE’s performance was reduced and the PIPs were also poorly calibrated (Supplementary Figs. 1–3, ‘random effects prior’ and ‘independent traits’ results). These results illustrate the benefits of having a flexible model that can adapt to different fine-mapping scenarios by learning effect-sharing patterns from the data (Supplementary Figs. 2 and 4–7). This flexibility comes at a computational cost—CAFEH was consistently faster than mvSuSiE (Fig. 2e and Supplementary Table 2)—but mvSuSiE was still fast enough to handle the largest fine-mapping datasets we considered.

We also compared mvSuSiE with CAFEH, PAINTOR and flashfm in a variety of simpler fine-mapping datasets simulated in a similar way to above but with only two traits (Supplementary Figs. 8–15). Even when the traits were simulated independently in accordance with PAINTOR’s modeling assumptions, PAINTOR had much lower power to detect causal SNPs than both SuSiE and mvSuSiE (Supplementary Fig. 8a). Both flashfm and mvSuSiE improved power over the SuSiE single-trait analyses, but mvSuSiE achieved much greater gains in power (Supplementary Figs. 8–15). mvSuSiE also had considerably lower computational cost than PAINTOR and flashfm (Supplementary Fig. 9 and Supplementary Table 2). The performance of CAFEH in these simpler simulations was similar to mvSuSiE except when the two traits were highly correlated (Supplementary Figs. 10 and 11).

In summary, these simulations demonstrate the benefits of mvSuSiE as an efficient and flexible multitrait fine-mapping method. In particular, mvSuSiE consistently increased power to detect causal SNPs, improved precision (reduced CS size) compared with fine-mapping each trait separately, and was the only method that provided both cross-trait and trait-wise significance measures.

### Multitrait fine-mapping of blood cell traits from UK Biobank

To illustrate mvSuSiE in a substantive application, we fine-mapped blood cell traits using data from the UK Biobank^37^. Previous analyses of these data include association analyses^38,39^ and single-trait fine-mapping^40,41^, but multitrait fine-mapping using mvSuSiE has the potential to improve power and precision of fine-mapping. Multitrait fine-mapping is also better for answering questions about shared genetic effects—which SNPs affect which traits—and hence provides insights into the underlying biology.

Focusing on a subset of 16 blood cell traits (Supplementary Table 3), we performed standard PLINK association analyses^42^ with *n* = 248,980 UK Biobank samples for which all 16 traits and imputed genotypes were available (Methods). We included covariates such as sex and age, as well as genotype principal components (PCs), to limit spurious associations due to population structure. From the results of these association analyses, we obtained 975 candidate genomic regions for fine-mapping (Supplementary Table 4). We then applied the mvSuSiE analysis pipeline to these 975 candidate regions (Methods). To understand the benefits of a multitrait fine-mapping approach, we also applied SuSiE to the same regions, separately for each trait.

### Genetic relationships among blood traits inform discovery of multitrait causal SNPs

From the 975 candidate regions, mvSuSiE identified 3,396 independent causal signals (95% cross-trait CSs). The median size of a CS was seven SNPs. Among these CSs, 726 contained just one SNP (‘1-SNP CS’); therefore, mvSuSiE identified 726 high-confidence candidate causal SNPs (PIP > 0.95; Supplementary Table 5). Several of these 1-SNP CSs (36) were not identified in any of our single-trait (SuSiE) analyses, underscoring the benefits of combining evidence across genetically related traits. Reassuringly, 496 of the 726 SNPs were also identified as high-confidence causal SNPs (PIP > 0.95) in the single-trait analyses reported in ref. ^41^, and 145 of 726 overlapped with those reported in ref. ^40^.

**Fig. 3:**
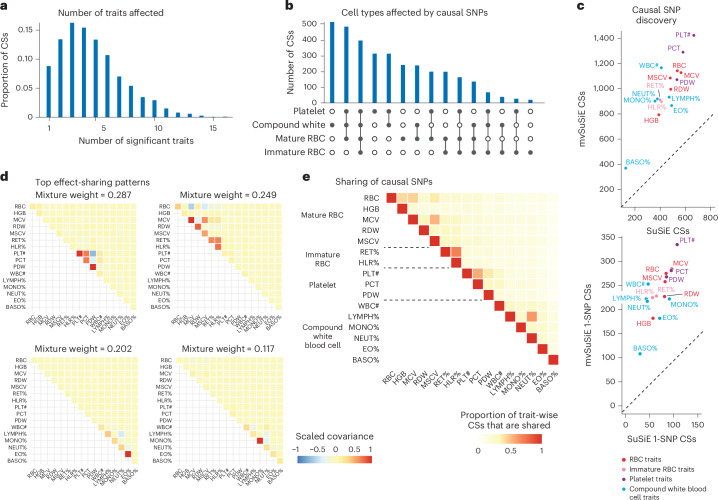
mvSuSiE fine-mapping and primary effect-sharing patterns in UK Biobank blood cell traits. **a**,**b**,**e**, Summaries of the 3,396 mvSuSiE CSs identified from the 975 candidate fine-mapping regions. **a**, Number of significant (‘average lfsr’ < 0.01) traits in each CS. **b**, Significant traits in CSs grouped by blood cell-type subsets. **e**, Pairwise sharing of significant CSs among the traits. In **e**, for each pair of traits, we show the ratio of the number of CSs that are significant in both traits to the number of CSs that are significant in at least one trait. **c**, Number of CSs and 1-SNP CSs for each trait identified by SuSiE and mvSuSiE (after removing CSs with purity less than 0.5). In **c**, each mvSuSiE count is the number of mvSuSiE CSs or 1-SNP CSs that are significant (‘average lfsr’ < 0.01) for the given trait. **d**, Covariance matrices in the mvSuSiE data-driven prior capturing the top sharing patterns (these are the covariance matrices with the largest mixture weights in the prior). The covariance matrices were scaled separately for each plot so that the plotted values lie between −1 and 1. See Supplementary Fig. 5 for the full set of 15 sharing patterns. PLT#, platelet count;MCV, mean corpuscular volume;PCT, relative volume of platelets;MSCV, mean sphered cell volume;WBC#, white blood cell count; PDW, platelet distribution width;RET%, reticulocyte percentage;HLR%, high light scatter reticulocytes percentage;RDW, red cell volume distribution width;NEUT%, neutrophil percentage;MONO%, monocyte percentage;LYMPH%, lymphocyte percentage; EO%, eosinophil percentage;HGB, hemoglobin concentration;BASO%, basophil percentage.

The number of CSs significant in each trait (‘average lfsr’ < 0.01) ranged from 370 (basophil percentage) to 1,423 (platelet count), and the number of 1-SNP CSs ranged from 108 to 335 (Fig. 3c). (Of note, 10 of 3,396 CSs were not significant in any traits at ‘average lfsr’ < 0.01.) Notably, mvSuSiE increased fine-mapping discovery and resolution compared to SuSiE single-trait fine-mapping—the number of trait-wise significant CSs increased, on average, 2.2-fold compared with SuSiE, and the number of trait-wise significant 1-SNP CSs increased, on average, 3.5-fold (Fig. 3c).

The fine-mapped SNPs from mvSuSiE were generally slightly more enriched for genomic regulatory annotations than those for SuSiE (Supplementary Fig. 16), providing indirect support for the additional mvSuSiE findings being driven by real signals rather than false positives. For example, the mvSuSiE-fine-mapped SNPs had an enrichment odds ratio of 11.9 for being an expression quantitative trait locus (eQTL) compared to 9.7 from SuSiE. We also analyzed enrichment of the fine-mapped SNPs for accessible chromatin regions in hematopoietic cell types^40^ (Supplementary Figs. 17 and 18 and Supplementary Tables 6 and 7). Similar to ref. ^41^, both the SuSiE and mvSuSiE results showed some of the expected enrichments, such as enrichment of SNPs affecting platelet-related traits for open chromatin in platelet-producing megakaryocytes.

mvSuSiE improved discovery and resolution over single-trait analysis by learning and exploiting patterns of shared (and not shared) genetic effects from the data. In these data, the most prominent learned patterns involved strong sharing of effects among traits for the same blood cell type (Fig. 3d). However, many other patterns were also identified (Supplementary Fig. 5), including both trait-specific and broad effects, suggesting that SNPs can affect blood cells in a wide variety of ways, presumably reflecting a wide variety of underlying biological mechanisms. By applying mvSuSiE with a prior that incorporates these learned sharing patterns, we obtained a genome-wide summary that underscores the diversity of genetic effects on blood cell traits (Fig. 3a,b,d). Genetic effects are more commonly shared among traits within the same blood cell type as one might expect (Fig. 3e), but SNPs affecting multiple blood cell types are also common (Fig. 3b).

### Multitrait fine-mapping reveals highly heterogeneous genetic determination of blood traits

**Fig. 4:**
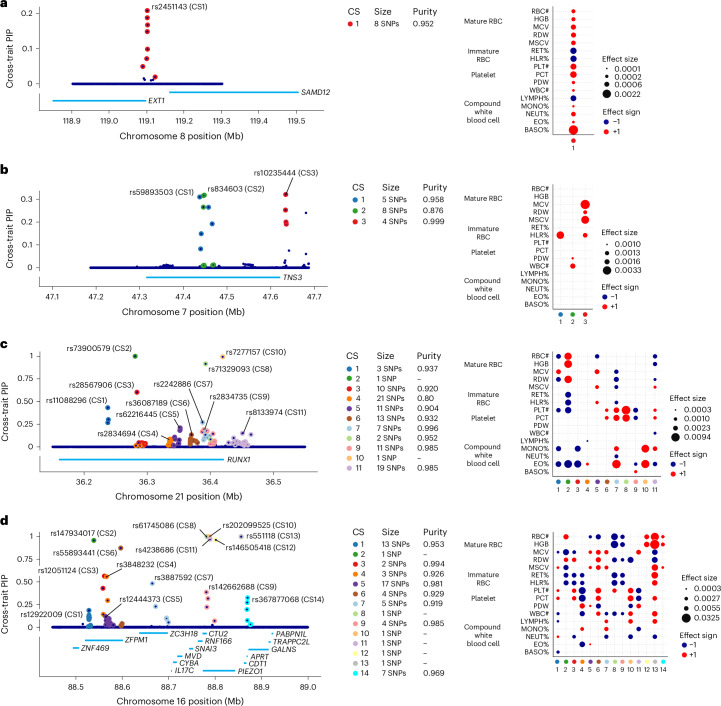
Examples of blood cell trait loci fine-mapped using mvSuSiE. **a**–**d**, Left, ‘PIP plots’ showing cross-trait PIPs for each SNP in the following fine-mapping regions: EXT1–SAMD12 locus (**a**), TNS3 locus (**b**), RUNX1 locus (**c**) and PIEZO1–ZFPM1 locus (**d**). The cross-trait PIP is an estimate of the probability that the SNP is causal for at least one trait. The labeled SNPs are the ‘sentinel SNPs’, the SNPs with the highest cross-trait PIP in each CS. ‘Purity’ is defined as the minimal absolute pairwise correlation (Pearson’s *r*) among SNPs in the CS. Right, plots showing the posterior effect estimates of the sentinel SNPs (only for CSs that are significant for the given trait, with ‘average lfsr’ <0.01). All estimates and tests are from a data sample of size *n* = 248,980.

To illustrate the potential for mvSuSiE to help dissect complex genetic association signals, we examine four example blood cell trait loci in more detail (Fig. 4).

Figure 4a shows the mvSuSiE results for the *EXT1–SAMD12* locus. Single-trait association analysis of this region shows only one trait, basophil percentage, with a genome-wide significant association (PLINK two-sided *t* test, *P* < 5 × 10^−8^). Similarly, single-trait fine-mapping with SuSiE identified a single CS for basophil percentage containing ten candidate SNPs, and no CSs for other traits. From these results, one might conclude that the causal SNP is specific to basophil percentage. However, the mvSuSiE fine-mapping results indicate that the CS is significant for most traits, suggesting that the causal SNP has broad effects across many traits. (Indeed, all traits had marginal association *P* values less than 0.003 with the lead SNP, which in some situations might be considered ‘significant’.) The mvSuSiE CS is smaller than the single-trait CS (eight versus ten SNPs), illustrating the improved fine-mapping resolution that can come from combining information across traits (Supplementary Fig. 19).

Figure 4b shows mvSuSiE results for the Tensin 3 locus. In ref. ^41^, the single-trait fine-mapping is used to identify causal signals for several RBC and white blood cell traits at this locus. However, a single-trait analysis does not tell us whether these signals are due to one or a few causal SNPs affecting many blood cell traits, or due to many causal SNPs affecting individual traits. The multitrait mvSuSiE analysis identified three causal signals (cross-trait CSs) with three distinct patterns of genetic effect—one mostly affects RBC traits (CS3); another has a detectable effect in HLR% only (CS1); and a third has smaller effects in both white blood cell and platelet traits (CS2). The three different patterns suggest that the biological effects of these SNPs are also different, and they suggest a multifaceted role for *TNS3* in affecting blood cell traits. This example illustrates the flexibility of mvSuSiE, including its ability to capture different patterns of effect sharing even within a single locus, and its ability to extract relatively simple inferences in quite complex situations.

Figure 4c shows a more complex example involving many signals in and around *RUNX1*. SNPs in the *RUNX1* locus have previously been associated with rheumatoid arthritis^43,44^ and other immune-related diseases (for example, refs. ^45,46^), and colocalization analyses have suggested that the causal SNPs are also associated with eosinophil proportions in blood^41^. Multitrait fine-mapping results from mvSuSiE suggest a complex picture with 11 signals (cross-trait CSs), each with detectable effects in many different blood cell traits, and some with no detectable effect on eosinophil proportions. These results suggest that the mechanisms by which this gene affects immune-related diseases may be more complex than just through eosinophils, possibly involving many platelet, RBC and other white blood cell traits.

Finally, Fig. 4d shows an even more complex example in which multiple causal signals are mapped to a region containing multiple genes, including *PIEZO1* and *ZFPM1*. This is a gene-dense region with well-studied connections to blood cell traits and blood-related diseases^47–51^. mvSuSiE identified 14 independent signals (cross-trait CSs) in the region. These 14 signals show a wide variety of effect patterns; for example, some are significant in only a few traits related to mature RBCs (for example, CS12, CS14), some are significant across a broader range of RBC traits (CS2) and some are significant across most traits (CS13). Regions of this level of complexity may take considerable additional investigation to fully understand. Although this is a complex example, we note that of the 14 CSs identified in this region, 7 contain a single SNP, demonstrating that even in complex regions, mvSuSiE can identify high-confidence causal SNPs.

## Discussion

We have introduced mvSuSiE, a fast and flexible multitrait fine-mapping method. mvSuSiE outperformed single-trait fine-mapping methods in both power and resolution. Unlike most available multitrait fine-mapping methods, mvSuSiE can efficiently analyze dozens of correlated traits and model complex patterns of effect-size variation using a flexible, data-driven prior distribution. The prior model also includes, as special cases, several simpler models that are commonly used in meta-analyses, such as the ‘fixed effects’ model that assumes equal effects in all traits, and the ‘random effects’ model that allows for different effect sizes among traits^52^. Although they may experience a potential loss of power, these models can be used in place of the data-driven models to speed-up computation, if desired. See the Supplementary Note for additional discussion, where we discuss some of mvSuSiE’s limitations, and give practical guidance on applying mvSuSiE to other types of traits (for example, binary traits) and on dealing with other potential complications (for example, missing data).

## Methods

### Ethics statement

This work used publicly available datasets, so ethical approval was not required.

### Multivariate multiple regression

mvSuSiE is based on a basic multivariate multiple regression model for *R* quantitative traits observed in *n* individuals,

2 $$Y \sim M{N}_{n\times R}(XB{,}{I}_{n}{,}V)$$

where $Y\in {{\mathbb{R}}}^{n\times R}$ is a matrix storing *n* observations of *R* traits, $X\in {{\mathbb{R}}}^{n\times J}$ is a matrix of *n* genotypes at *J* SNPs, $B \in {{\mathbb{R}}}^{J\times R}$ is a matrix of regression coefficients (‘effects’) for the *J* SNPs and *R* traits, *V* is an *R* × *R* covariance matrix (we assume *V* is invertible), ${I}_{n}$ is the *n* × *n* identity matrix and $M{N}_{n\times R}\left(M,{\Sigma }^{\mathrm{row}},{\Sigma }^{\mathrm{col}}\right)$ denotes the matrix normal distribution^54,55^ with mean $M\in {{\mathbb{R}}}^{n\times R}$ and covariance matrices ${\Sigma }^{\mathrm{row}}$ and ${\Sigma }^{\mathrm{col}}$ (of dimensions *n* × *n* and *R* × *R*, respectively).

### Intercept

We do not explicitly include an intercept in equation (2). Instead, we account for an intercept implicitly by ‘centering’ the columns of *X* and the columns of *Y* so that the mean of each column is zero. From a Bayesian perspective, centering the columns of *X* and *Y* is equivalent to integrating with respect to an (improper) uniform prior on the intercept. (This is a multivariate generalization of the result for univariate regression in ref. ^56^. See the Supplementary Note for a more formal proof of this result.) In short, centering eliminates the need to explicitly include an intercept in equation (2), and we proceed with mvSuSiE assuming that *X* and *Y* have been centered.

### The mvSuSiE model

mvSuSiE generalizes the SuSiE model^13^ to the multivariate setting:

3 $$\begin{array}{l}B=\mathop{\sum }\limits_{l=1}^{L}{B}^{(l)}\\ {B}^{(l)}={{\boldsymbol{\gamma }}}^{(l)}\otimes {{\bf{b}}}^{(l)}\end{array}$$

where ${{\boldsymbol{\gamma }}}^{\left(l\right)}\in {\left(\mathrm{0,1}\right)}^{J}$ is a vector of indicator variables in which exactly one of the *J* elements is one and the remaining are zero, ${{\bf{b}}}^{\left(l\right)}\in {{\mathbb{R}}}^{R}$ is a vector of regression coefficients and ${\bf{u}}\otimes {\bf{v}}={{\bf{uv}}}^{{\rm{\top }}}$ denotes the outer product of (column) vectors **u** and **v**. The coefficients *B* defined in this way are a sum of *L* ‘single effects’ ${B}^{\left(l\right)}$. In particular, matrix ${B}^{\left(l\right)}\in {{\mathbb{R}}}^{J\times R}$ has at most one row containing nonzero values, and these nonzero values are determined by ${{\bf{b}}}^{\left(l\right)}$. We therefore refer to ${B}^{\left(l\right)}$ as a ‘single-effect matrix’ because it encodes the effects of a single SNP. The final set of coefficients *B* is a matrix with at most *L* rows containing nonzero values.

Similar to SuSiE, we introduce priors for the indicator variables ${{\boldsymbol{\gamma }}}^{\left(l\right)}$ and regression coefficients ${{\bf{b}}}^{\left(l\right)}$,

4 $$\begin{array}{l}{{\boldsymbol{\gamma }}}^{\left(l\right)}\sim \mathrm{Multinom}\left(1,\boldsymbol{\pi} \right)\\ {{\bf{b}}}^{\left(l\right)}\sim {g}_{l}\end{array}$$

where $\mathrm{Multinom}\left(m,\pi \right)$ denotes the multinomial distribution for *m* random multinomial trials with category probabilities $\boldsymbol{\pi} =\left({\pi }_{1},\ldots ,{\pi }_{J}\right)$, such that ${\pi }_{j}\ge 0$ and ${\sum }_{j=1}^{J}{\pi }_{j}=1$. The ${\pi }_{j}$’s are the prior inclusion probabilities. By default, we assume a uniform prior; that is, ${\pi }_{j}=1/J$, for $j=1,\ldots ,J$. (All the results in this paper use this default prior.) Our software implementation of mvSuSiE also supports for other choices of $\boldsymbol{\pi}$; for example, $\boldsymbol{\pi}$ could be determined by external biological information about the SNPs (for example, ref. ^57^).

The prior distribution ${g}_{l}$ for each single effect ${{\bf{b}}}^{\left(l\right)}$ should capture the variety of effect-sharing patterns we expect from the multiple traits. To this end, we use a prior similar to the mixture of multivariate normals prior introduced in ref. ^31^,

5 $${g}_{l}\left({\bf{b}}\right)=\mathop{\sum }\limits_{k=1}^{K}\omega_k N_R\left({\bf{b}};\boldsymbol{0},{\sigma }_{0l}^{2}{U}_{k}\right)$$

where each ${U}_{k}$ is a (possibly singular) covariance matrix, ${\sigma }_{0l}^{2}\ge 0$ scales the prior for each single effect *l*, ${\boldsymbol{\omega }}=\left(\omega_1,\ldots ,\omega_K\right)$ is a vector of mixture weights, such that $\omega_k\ge 0$, ${\sum }_{k=1}^{K}{\omega }_{k}=1$ and $N_d\left(\boldsymbol{x};\boldsymbol{\mu},\Sigma \right)$ denotes the multivariate normal distribution on ${\boldsymbol{x}}\in {{\mathbb{R}}}^{d}$ with mean $\boldsymbol{\mu} \in {{\mathbb{R}}}^{d}$ and *d* × *d* covariance $\Sigma$. The covariance matrices ${\mathscr{U}}=({{U}}_{{1}}{,}{\ldots }{,}{{U}}_{{K}})$ and the mixture weights $\omega$ must be chosen beforehand, whereas the prior scaling parameters ${\sigma }_{01}^{2},\ldots ,{\sigma }_{0L}^{2}$ are treated as unknown, and are estimated from the data.

In summary, mvSuSiE is a multivariate regression model with a flexible mixture-of-normals prior on the ‘single effects’ ${\bf{b}}^{\left(l\right)}$. The unknowns of primary interest are the single-effect matrices ${B}^{\left(l\right)}$. As we explain in more detail below, we compute a posterior distribution of the single effects, which is then used to compute key fine-mapping statistics, including PIPs and CSs. The scaling factors ${\sigma }_{0l}^{2}$ are not of primary interest to the fine-mapping (‘nuisance parameters’), and are estimated from the data to aid in better posterior estimation of the single effects. Other model parameters, such as the residual covariance matrix *V*, are assumed to be known or should have been estimated previously. In the Supplementary Note, we give guidance on choosing these parameters or estimating them from data. See also the Supplementary Note for derivations, development of the model fitting algorithm and additional technical details.

### UK Biobank data

The UK Biobank is a prospective cohort study with detailed phenotype and genotype data collected from approximately 500,000 participants recruited in the United Kingdom, with ages of between 40 and 69 years at the time of recruitment^37,58^. For fine-mapping, we focused on a subset of 16 blood cell traits from the UK Biobank hematology data collection^59^. These blood cell traits were also the focus of a recent association analysis^38,39^ and fine-mapping studies^40,41^. Several of the UK Biobank blood cell traits are based on the same measured quantities and are therefore highly correlated; therefore, we did not include all blood cell traits in our analyses. For example, relative volume of erythrocytes, also known as ‘hematocrit’ (HCT), is calculated from mean corpuscular volume and RBC count (RBC#), so to avoid including highly correlated traits we did not include HCT. The blood cell traits used in our fine-mapping analyses are summarized in Supplementary Table 3.

The UK Biobank imputed genotypes feature a high density of SNPs, making them well suited for fine-mapping. We used a subset of the 502,492 available UK Biobank genotypes (version 3), removing samples that met one or more of the following criteria for exclusion: mismatch between self-reported and genetic sex; pregnant; one or more data entries needed for the analysis or data preparation steps are missing; and, following refs. ^38,41^, a blood-related disease was reported in the hospital in-patient data (blood-related diseases included leukemia, lymphoma, bone marrow transplant, chemotherapy, myelodysplastic syndrome, anemia, HIV, end-stage kidney disease, dialysis, cirrhosis, multiple myeloma, lymphocytic leukemia, myeloid leukemia, polycythemia vera and hemochromatosis). Additionally, we excluded outlying genotype samples based on heterozygosity and/or rate of missing genotypes as defined by UK Biobank (data field 22027), and we removed any individuals having at least one relative in the cohort based on UK Biobank kinship calculations (samples with a value other than zero in data field 22021). Finally, to limit confounding due to population structure, we included only genotype samples marked as ‘White British’ (based on a PCs analysis of the genotypes^37^ stored in data field 22009). After filtering genotype samples according to these criteria, 257,605 samples remained.

We applied quantile normalization to the 16 blood cell traits measured in the 257,605 samples, separately for each trait, to transform each trait to the standard normal distribution. Because ultimately we aimed to jointly model the 16 blood cell traits, we removed outlying phenotypes according to a simple multivariate normal distribution of the phenotypes. Specifically, after quantile normalization, we measured the Mahalanobis distance ${{\bf{y}}}_{i}^{{\rm{\top }}}\hat{\Sigma }^{-1}{{\bf{y}}}_{i}$ for each individual *i*, where ${{\bf{y}}}_{i}$ is the vector of 16 blood cell traits measured in individual *i*, and $\hat{\Sigma }$ is the sample covariance matrix estimated from the 257,605 UK Biobank samples. We discarded samples with Mahalanobis distance falling within the (0.99, 1) quantile of the chi-square distribution with 16 degrees of freedom. This step removed 8,625 samples, for a final total of 248,980 UK Biobank samples.

Base-pair positions of the SNPs are reported using the Genome Reference Consortium human genome assembly 37 (hg19).

### Association analyses of UK Biobank blood cell traits

Using the UK Biobank genotype and phenotype data prepared as described above, we computed association statistics for each of the 16 blood cell traits and for all available biallelic SNPs on autosomal chromosomes meeting the following criteria: minor allele frequency of 0.1% or greater; information (‘INFO’) score of 0.6 or greater (the INFO score quantifies imputation quality). The same criteria were used in ref. ^60^ to filter the SNPs.

Association statistics were computed using the --glm function in PLINK (version 2.00a2LM, 64-bit Intel, 21 February 2009)^42^ with hide-covar no-x-sex omit-ref –vif 100. Following refs. ^1,41^, we included the following covariates in the association analyses: sex (data field 31), age at recruitment (data field 21022), age × age, assessment center (data field 54) and genotype measurement batch (data field 22000). To limit inflation of spurious associations due to population structure, we also included the top ten genotype PCs as covariates following the previous association analyses of UK Biobank data (for example, ref. ^61^). (These PCs were previously computed by UK Biobank^37^ and stored in data field 22009.) The covariates input file for PLINK was prepared by calling the ‘model.matrix’ function in R and standardizing quantitative covariates (age, PCs) to have a mean of 0 and a variance of 1.

The summary data provided as input to SuSiE and mvSuSiE were the *z* scores and *P* values extracted from the T_STAT and P columns in the plink2 --glm outputs. The association statistics computed using PLINK are available in a Zenodo repository (Data availability).

### Selection of regions for fine-mapping

To select regions for fine-mapping, we adapted the approach used in ref. ^41^ to the multivariate setting. In brief, we began by identifying regions for each trait separately. For each significant association (PLINK two-sided *t* test, *P* *<* 5 × 10^−8^), we defined the fine-mapping region as all SNPs within ±250 kb of the significant association. Next, any regions overlapping by one or more SNPs were combined into a larger region. We repeated the process of combining regions until no regions overlapped. This resulted in a set of fine-mapping regions for each of the 16 blood cell traits, similar to ref. ^41^. To form a single set of fine-mapping regions for all 16 traits, we then merged two regions from different traits whenever they overlapped. The end result of this procedure was a set of 975 disjoint fine-mapping regions satisfying the following two properties: (1) all significant SNPs (with PLINK two-sided *t* test, *P* *<* 5 × 10^−8^) belong to exactly one region; and (2) all SNPs within 250 kb of a significant SNP belong to exactly one region. This procedure generated fine-mapping regions that varied considerably in size—their lengths ranged from 411 kb to 8.73 Mb (average size = 961 kb; median size = 686 kb); and the number of SNPs ranged from 93 SNPs to 36,605 SNPs (average number of SNPs = 4,776; median number of SNPs = 3,514). A listing of all 975 regions is given in Supplementary Table 4. These same regions were used in both the single-trait and multitrait fine-mapping.

Note that we did not fine-map the extended MHC^62^ (defined as base-pair positions of 25–36 Mb on chromosome 6). The MHC is particularly challenging to analyze and interpret, and therefore is typically analyzed separately^63–65^.

### Simulations using UK Biobank genotypes

We evaluated the fine-mapping methods on datasets generated using real genotypes *X* and simulated phenotypes *Y*. For the genotypes, we used the UK Biobank imputed genotypes. We simulated *Y* from different mvSuSiE models (see below). The genotype data were curated following the data preparation steps described above, so *n* = 248,980 in all our simulations. (To clarify, these data preparation steps included removing outlying blood cell trait observations. Although this particular filtering step was not needed as we did not use the UK Biobank phenotype data in the simulations, for convenience, we used the data prepared with this filtering step.)

### Simulation scenarios

We implemented three fine-mapping scenarios in the simulations.

In the simplest simulations, which we used to compare all of the methods (SuSiE, mvSuSiE, CAFEH, PAINTOR and flashfm), we simulated two traits under the following three different scenarios: (1) independent traits with independent effects; (2) independent traits with correlated effects; and (3) correlated traits with independent effects. This simpler scenario was intended mainly for comparisons with PAINTOR and flashfm so as not to unfairly disadvantage these methods; flashfm cannot handle a large number of traits, and PAINTOR cannot handle a large number of causal SNPs, and assumes independent traits and independent effects (Table 1). However, for completeness, we also compared with SuSiE and CAFEH in this simulation scenario.

For comparing other fine-mapping methods (mvSuSiE, SuSiE, CAFEH), we simulated datasets under two more complex scenarios, which we refer to as ‘scenario A’ and ‘scenario B’.

In scenario A, we simulated 20 independent traits in which the SNP effects were either specific to one trait or shared among traits in simple ways (equal effects among 2 traits, equal effects among half of the traits or correlated equally among all 20 traits). In the results, we call scenario A the ‘trait-specific + shared effects’ scenario.

Scenario B was intended to capture a combination of factors that one might more realistically encounter in fine-mapping studies. It is also more challenging because the traits are correlated and their effects are shared in complex ways. Specifically, we simulated using a residual covariance matrix *V* and sharing patterns ${U}_{k}$ obtained from our analyses of the UK Biobank blood cell traits. In the results, we refer to scenario B as the ‘complex shared effects’ scenario.

The steps taken to simulate the datasets are given in the Supplementary Note.

### Details of the methods compared

In this section, we describe how we ran the methods on the simulated datasets. SuSiE, mvSuSiE and PAINTOR were run using the *z* scores and the in-sample LD. CAFEH and flashfm were run using the effect estimates, standard errors of these effect estimates and in-sample LD. Some methods, including mvSuSiE, also accepted an additional input, the sample size (*n*), in which case we provided this as well. flashfm also required the reference allele frequencies, which in all our analyses were the minor allele frequencies.

### PAINTOR

We ran PAINTOR^19^ in only the two-trait simulations. PAINTOR was designed to work with functional genomic annotation data, so to run PAINTOR, we created a single ‘dummy’ annotation in which all SNPs were assigned to this annotation (that is, all entries of the annotation matrix were set to 1). For all datasets, we asked PAINTOR to enumerate all possible configurations up to two causal SNPs. (In the two-trait simulations, the true number of causal SNPs was always 2.) We did not use the ‘mcmc’ option (-mcmc) because the outputted PIPs, when using this option, were all zero in our tests. (The same issue was reported at https://github.com/gkichaev/PAINTOR_V3.0/issues/5.) All other PAINTOR options were kept at their default settings. Of note, PAINTOR does not accept *n* (the sample size) as input. Also note that PAINTOR assumes that both traits and effects are independent across traits (Table 1).

### flashfm

We ran flashfm^20^ in only the two-trait simulations. We ran flashfm by calling function FLASHFMwithFINEMAP from R package flashfm (version 0.0.0.9000). This function internally calls FINEMAP^10^ (version 1.4.1) with settings --sss --n-configs-top 1000 --n-causal-snps 10, which allows configurations of up to ten causal SNPs. We ran flashfm with four CPUs (NCORES = 4). All other flashfm settings were kept at their defaults. The inputs to ‘FLASHFMwithFINEMAP’ were the effect estimates, the standard errors of these effect estimates, minor allele frequencies, vector of trait means and sample size *n*. Because *Y* was centered and standardized in the simulations, the vector of trait means was simply a vector of zeros of length *R*.

### CAFEH

We used the fit_cafeh_summary interface in CAFEH 1.0 (ref. ^23^) installed with Python 3.7.4. The fit_cafeh_summary function accepts the following data inputs: effect estimates, standard errors of those estimates, LD matrix and sample size *n*. When calling fit_cafeh_summary, all optional arguments were kept at the software defaults. CAFEH’s default setting for the upper limit on the number of single effects (‘*K*’ in the CAFEH model) is 10, which is the same default in SuSiE and mvSuSiE. Of note, CAFEH assumes that traits and effects are independent across traits (Table 1). CAFEH outputs CSs without any filter on CS purity. Therefore, to make the CAFEH CSs comparable to the SuSiE and mvSuSiE CSs, we excluded CSs with purity <0.5. For assessing the performance of CAFEH PIPs and trait-wise PIPs (in CAFEH, these are called ‘study PIPs’), we called get_pip and get_study_pip.

Of note, the two CAFEH summary data interfaces—fit_cafeh_summary and fit_cafeh_z—produce the same or very similar results when *X* is standardized. Both functions internally call the function ‘CAFEHSummary’ with the same LD matrix, but provide different effect estimates and standard errors of the effect estimates. Let $\hat{{\boldsymbol{\beta }}}$ denote the vector of effect estimates (with one entry per SNP) and let $\hat{{\bf{s}}}$ denote the vector of standard errors (also with one entry per SNP). If fit_cafeh_summary calls CAFEHSummary with inputs $\hat{{\boldsymbol{\beta }}},\,\hat{{\bf{s}}}$, and assuming *X* is standardized, then it can be shown that fit_cafeh_z calls CAFEHSummary with inputs $\sqrt{n}\hat{{\boldsymbol{\beta }}},\,\sqrt{n}\hat{{\bf{s}}}$. Because CAFEHSummary is invariant to rescaling of $\hat{{\boldsymbol{\beta }}},\,\hat{{\bf{s}}}$—that is, CAFEHSummary generates the same result with inputs $a\hat{{\boldsymbol{\beta }}}$ and $a\hat{{\bf{s}}}$ for any choice of scalar $a > 0$—it follows that fit_cafeh_summary and fit_cafeh_z also produce the same result when *X* is standardized. In practice, this invariance does not hold exactly as it requires that the prior on the effects also be appropriately rescaled, but empirically we have found that the CAFEH PIPs and posterior effect estimates are almost the same for different choices of $a > 0$ (https://github.com/karltayeb/cafeh/blob/current_working_branch/notebooks/CAFEHS_scale_invariance.ipynb).

### SuSiE

We ran SuSiE by calling the function susie_rss from susieR^13^ (version 0.12.12). In each dataset, we ran susie_rss once per trait. The susie_rss interface accepts different types of summary data; we provided *z* scores, in-sample LD and sample size *n*. For all simulations, we set *L*, the maximum number of nonzero effects, to 10. (We also set *L* = 10 for the two-trait simulations, although there were never more than two causal SNPs in these simulations.) We estimated the residual variance (estimate_residual_variance = TRUE), which is the recommended setting when the LD is estimated from the ‘in-sample’ data. We set the maximum number of IBSS iterations to 1,000 (max_iter = 1000). The remaining optional arguments were kept at their defaults.

Because SuSiE analyzes each trait separately, it does not provide direct evidence that a SNP is a cross-trait causal SNP. To quantify performance in this task and compare with mvSuSiE, we quantified the evidence for a cross-trait causal SNP using an ad hoc metric, the ‘maximum PIP’, defined as

6 $${\mathrm{max-PIP}}_{j}:=\mathop{\max }\limits_{r\in \left(1,\ldots ,R\right)}{\mathrm{PIP}}_{{jr}}$$

where ${\mathrm{PIP}}_{{jr}}$ is the PIP for SNP *j* obtained from the SuSiE analysis of trait *r*.

### mvSuSiE

We ran mvSuSiE using the mvsusie_rss interface from mvsusieR (version 0.0.3.0518, git commit id 9f28916). While susie_rss accepts a ‘vector’ of *z* scores, mvsusie_rss accepts a ‘matrix’ of *z* scores (specifically, a *J* × *R* matrix). In the simulations, we compared several mvSuSiE variants using different prior choices; for more details, see the Supplementary Note. (In the two-trait simulations, we only used the canonical prior. This was a mixture of multivariate normals with *K* = 7 components.) We also compared mvSuSiE with different settings of the residual covariance *V* (Supplementary Note). In all cases, we ran mvsusie_rss with the following settings: L = 10, max_iter = 1000, estimate_prior_variance = TRUE, estimate_prior_method = ‘EM’, precompute_covariances = TRUE and n_thread = 4. (We set *L* = 10 for the two-trait simulations, although there were never more than two causal SNPs in these simulations.) All other options were kept at the default settings.

### Computing environment

All analyses of the simulated datasets were run on Linux machines (Scientific Linux 7.4) with 4 Intel Xeon E5-2680v4 (‘Broadwell’) processors, and with R (version 4.1.0) linked to the OpenBLAS (version 0.3.13) optimized numerical libraries. At most 10 GB of memory was needed to perform a fine-mapping analysis of a single simulated dataset using one of the methods. We used DSC (version 0.4.3.5) to perform the simulations.

### Fine-mapping of UK Biobank blood cell traits using SuSiE and mvSuSiE

We fit an mvSuSiE model to each fine-mapping dataset—specifically, the *z* scores matrix *Z* and LD matrix *R*^66^—by calling mvsusie_rss from the mvsusieR package with the following settings: L = 10, N = 248980, precompute_covariances = TRUE, estimate_prior_variance = TRUE, estimate_prior_method = ‘EM’, max_iter = 1000 and n_thread = 1. We ran SuSiE on *Z*, *R* separately for each trait (that is, column of *Z*) by calling susie_rss with the following settings: n = 248980, L = 10, max_iter = 1000, estimate_prior_variance = TRUE, refine = TRUE. Any CSs returned by susie_rss or mvsusie_rss with purity less than 0.5 were removed.

### Enrichment analysis of regulatory annotations using GREGOR

We performed enrichment analyses of the SuSiE and mvSuSiE blood cell trait fine-mapping results using GREGOR^67^ (version 1.4.0). In brief, GREGOR performs an enrichment analysis for a ‘positive set’ of SNPs by calculating overlap with the given regulatory annotation, then estimates the probability of the observed overlap against its expectation using a set of ‘matched control SNPs’. We ran GREGOR with the following settings: pop = ‘EUR’, r2_threshold = 0.7, ld_window_size = 10000, min_neighbor = 10, job_number = 10.

Although GREGOR provides *P* values, we found some issues with these *P* values (for example, some exceeded 1). Therefore, for each annotation, we extracted the intermediate GREGOR outputs to get a 2 × 2 table of the SNP counts of inside and outside the annotation intersected with positive set and matched control set. We then used this 2 × 2 table to perform Fisher′s exact test and this was the final *P* value reported. Additional details about the GREGOR analysis are provided in 20231106_GREGOR_functional_enrichment.ipynb Jupyter notebook in one of the Zenodo repositories.

We assessed enrichment for a total of 19 regulatory genomic annotations, including enhancer–promoter regions and transcription factor binding sites^68^; genomic structural elements^69^; eQTLs in multiple tissues (based on different FDRs)^70^; RNA polymerase II binding in EPC-treated human embryonic stem cells^71^; and binding intervals for specific transcription factors. The specific transcription factors included were promyelocytic leukemia zinc finger protein, FOSL2, NR2F2 and FOXO1 (ref. ^71^). The BED annotation files are included in one of the Zenodo repositories.

Using these regulatory genomic annotations, we performed two sets of GREGOR enrichment analyses, one using the SuSiE fine-mapping results and another set using the mvSuSiE fine-mapping results. We performed these enrichment analyses separately for the fine-mapping results for each blood cell trait, as well as the ‘global’ (cross-trait) results. For mvSuSiE, we included a SNP in the cross-trait positive set if the SNP was included in at least one 95% CS and/or the global PIP was greater than 0.7. We included a SNP in the positive set for a given blood cell trait if the SNP was included in at least one 95% CS and the lfsr for the given trait was less than 0.01.

For SuSiE, we included a SNP in the positive set for a given trait if the SNP was included in at least one 95% CS and/or the PIP was greater than 0.7. The SuSiE cross-trait positive set was defined as the union of the 16 positive sets from the SuSiE analyses of each of the 16 traits.

### Enrichment analysis of hematopoietic cell types using gchromVAR

We performed additional enrichment analyses of the SuSiE and mvSuSiE blood cell trait fine-mapping results using gchromVAR^40^. In brief, gchromVAR assesses overlap of the fine-mapped SNPs and regions of accessible chromatin, separately in different hematopoietic cell types. (This is actually a weighted overlap in which we have defined the weights as the SuSiE or mvSuSiE PIPs.) Each enrichment analysis was performed according to the steps described in the gchromVAR R package vignette (version 0.3.2). The *z* scores returned by the computeWeightedDeviations function were then refined using adaptive shrinkage implemented in ashr^34^ (version 2.2-57). The adaptive shrinkage posterior *z* scores (posterior means divided by posterior s.d.) and lfsr values were used to report the final enrichment results.

Similar to the GREGOR enrichment analyses, we performed two separate enrichment analyses with gchromVAR, one using the SuSiE results and another using the mvSuSiE results. For SuSiE, we included a SNP for a given trait if the PIP > 0.01 for that trait. A total of 100,090 SNPs had PIP > 0.01 in at least one of the blood cell traits. For mvSuSiE, we included a SNP for a given trait if the global PIP > 0.01 and if the CS was significant for the given trait (lfsr < 0.01). A total of 39,884 SNPs had a global PIP > 0.01.

### Reporting summary

Further information on research design is available in the Nature Portfolio Reporting Summary linked to this article.

## Online content

Any methods, additional references, Nature Portfolio reporting summaries, source data, extended data, supplementary information, acknowledgements, peer review information; details of author contributions and competing interests; and statements of data and code availability are available at 10.1038/s41588-025-02486-7.

## Supplementary information

Supplementary Information Supplementary Note and Figs. 1–19.

Reporting Summary

Peer Review File

Supplementary Tables Supplementary Tables 1–8.

## Data Availability

UK Biobank data are available at https://www.ukbiobank.ac.uk; PLINK association test statistics from the UK Biobank blood cell traits are available at 10.5281/zenodo.8088040 (ref. ^72^).

## Code availability

The mvsusieR R package implementing our methods is available on GitHub at https://github.com/stephenslab/mvsusieR; mvsusieR 0.1.8 is also available on Zenodo (10.5281/zenodo.17296669)^73^. Additional code resources accompanying this paper include the following: code for preparing the UK Biobank data, 10.5281/zenodo.8400278 (ref. ^74^); code for performing the fine-mapping simulations, 10.5281/zenodo.8087907 (ref. ^75^); additional code for the fine-mapping simulations and the fine-mapping analyses of the UK Biobank blood cell traits, 10.5281/zenodo.8094982 (ref. ^76^). Other R packages and software used in this work include the following: susieR 0.12.12, https://github.com/stephenslab/susieR; mashr 0.2.59, https://github.com/stephenslab/mashr; flashr 0.6-8, https://github.com/stephenslab/flashr; PAINTOR 3.1, https://github.com/gkichaev/PAINTOR_V3.0; BayesSUR 2.0-1, https://cran.r-project.org/package=BayesSUR; flashfm 0.0.0.9000, https://github.com/jennasimit/flashfm; FINEMAP 1.4.1, http://www.christianbenner.com; msCAVIAR 0.1, https://github.com/nlapier2/MsCAVIAR; CAFEH 1.0, https://github.com/karltayeb/cafeh; hyprcoloc 1.0, https://github.com/cnfoley/hyprcoloc; moloc 0.1.0, https://github.com/clagiamba/moloc; MFM 0.2-1, https://github.com/jennasimit/MFM; gchromVAR 0.3.2, https://github.com/caleblareau/gchromVAR; GREGOR 1.4.0, http://csg.sph.umich.edu/GREGOR/; R 4.1.0, https://cran.r-project.org; Python 3.7.4, https://www.python.org; PLINK 2.00a2LM, 64-581 bit Intel, 21 February 2009, https://www.cog-genomics.org/plink2; LDstore 1.1, http://christianbenner.com; DSC 0.4.3.5, https://github.com/stephenslab/dsc.

## References

[1] Canela-Xandri, O., Rawlik, K. & Tenesa, A. An atlas of genetic associations in UK Biobank. *Nat. Genet.* **50**, 1593–1599 (2018). 30349118 10.1038/s41588-018-0248-z PMC6707814

[2] Visscher, P. M. et al. 10 years of GWAS discovery: biology, function, and translation. *Am. J. Hum. Genet.* **101**, 5–22 (2017). 28686856 10.1016/j.ajhg.2017.06.005 PMC5501872

[3] Buniello, A. et al. The NHGRI-EBI GWAS catalog of published genome-wide association studies, targeted arrays and summary statistics 2019. *Nucleic Acids Res.* **47**, D1005–D1012 (2018). 10.1093/nar/gky1120 PMC6323933 30445434

[4] Tam, V. et al. Benefits and limitations of genome-wide association studies. *Nat. Rev. Genet.* **20**, 467–484 (2019). 31068683 10.1038/s41576-019-0127-1

[5] Hormozdiari, F., Kostem, E., Kang, E. Y., Pasaniuc, B. & Eskin, E. Identifying causal variants at loci with multiple signals of association. *Genetics* **198**, 497–508 (2014). 25104515 10.1534/genetics.114.167908 PMC4196608

[6] Kichaev, G. et al. Integrating functional data to prioritize causal variants in statistical fine-mapping studies. *PLoS Genet.* **10**, e1004722 (2014). 25357204 10.1371/journal.pgen.1004722 PMC4214605

[7] Maller, J. B. et al. Bayesian refinement of association signals for 14 loci in 3 common diseases. *Nat. Genet.* **44**, 1294–1301 (2012). 23104008 10.1038/ng.2435 PMC3791416

[8] Yang, J. et al. Conditional and joint multiple-SNP analysis of GWAS summary statistics identifies additional variants influencing complex traits. *Nat. Genet.* **44**, 369–375 (2012). 22426310 10.1038/ng.2213 PMC3593158

[9] Chen, W. et al. Fine mapping causal variants with an approximate Bayesian method using marginal test statistics. *Genetics* **200**, 719–736 (2015). 25948564 10.1534/genetics.115.176107 PMC4512539

[10] Benner, C. et al. FINEMAP: efficient variable selection using summary data from genome-wide association studies. *Bioinformatics* **32**, 1493–1501 (2016). 26773131 10.1093/bioinformatics/btw018 PMC4866522

[11] Wen, X., Lee, Y., Luca, F. & Pique-Regi, R. Efficient integrative multi-SNP association analysis via deterministic approximation of posteriors. *Am. J. Hum. Genet.* **98**, 1114–1129 (2016). 27236919 10.1016/j.ajhg.2016.03.029 PMC4908152

[12] Lee, Y., Luca, F., Pique-Regi, R. & Wen, X. Bayesian multi-SNP genetic association analysis: control of FDR and use of summary statistics. Preprint at *bioRxiv*10.1101/316471 (2018).

[13] Wang, G., Sarkar, A., Carbonetto, P. & Stephens, M. A simple new approach to variable selection in regression, with application to genetic fine mapping. *J. R. Stat. Soc. B* **82**, 1273–1300 (2020). 10.1111/rssb.12388 PMC10201948 37220626

[14] Wallace, C. et al. Dissection of a complex disease susceptibility region using a Bayesian stochastic search approach to fine mapping. *PLoS Genet.* **11**, e1005272 (2015). 26106896 10.1371/journal.pgen.1005272 PMC4481316

[15] Schaid, D. J., Chen, W. & Larson, N. B. From genome-wide associations to candidate causal variants by statistical fine-mapping. *Nat. Rev. Genet.* **19**, 491–504 (2018). 29844615 10.1038/s41576-018-0016-z PMC6050137

[16] Zou, Y., Carbonetto, P., Wang, G. & Stephens, M. Fine-mapping from summary data with the ‘Sum of Single Effects’ model. *PLoS Genet.* **18**, e1010299 (2022). 35853082 10.1371/journal.pgen.1010299 PMC9337707

[17] Stephens, M. A unified framework for association analysis with multiple related phenotypes. *PLoS ONE* **8**, e65245 (2013). 23861737 10.1371/journal.pone.0065245 PMC3702528

[18] Lewin, A. et al. MT-HESS: an efficient Bayesian approach for simultaneous association detection in OMICS datasets, with application to eQTL mapping in multiple tissues. *Bioinformatics* **32**, 523–532 (2016). 26504141 10.1093/bioinformatics/btv568 PMC4743623

[19] Kichaev, G. et al. Improved methods for multi-trait fine mapping of pleiotropic risk loci. *Bioinformatics* **33**, 248–255 (2017). 27663501 10.1093/bioinformatics/btw615 PMC5254076

[20] Hernández, N. et al. The flashfm approach for fine-mapping multiple quantitative traits. *Nat. Commun.* **12**, 6147 (2021). 34686674 10.1038/s41467-021-26364-y PMC8536717

[21] Zhao, Z. et al. BayesSUR: an R package for high-dimensional multivariate Bayesian variable and covariance selection in linear regression. *J. Stat. Softw.* **100**, 1–32 (2021).

[22] LaPierre, N. et al. Identifying causal variants by fine mapping across multiple studies. *PLoS Genet.* **17**, e1009733 (2021). 34543273 10.1371/journal.pgen.1009733 PMC8491908

[23] Arvanitis, M., Tayeb, K., Strober, B. J. & Battle, A. Redefining tissue specificity of genetic regulation of gene expression in the presence of allelic heterogeneity. *Am. J. Hum. Genet.* **109**, 223–239 (2022). 35085493 10.1016/j.ajhg.2022.01.002 PMC8874223

[24] Asimit, J. L. et al. Stochastic search and joint fine-mapping increases accuracy and identifies previously unreported associations in immune-mediated diseases. *Nat. Commun.* **10**, 3216 (2019). 31324808 10.1038/s41467-019-11271-0 PMC6642100

[25] Foley, C. N. et al. A fast and efficient colocalization algorithm for identifying shared genetic risk factors across multiple traits. *Nat. Commun.* **12**, 764 (2021). 33536417 10.1038/s41467-020-20885-8 PMC7858636

[26] Giambartolomei, C. et al. A Bayesian framework for multiple trait colocalization from summary association statistics. *Bioinformatics* **34**, 2538–2545 (2018). 29579179 10.1093/bioinformatics/bty147 PMC6061859

[27] Wallace, C. A more accurate method for colocalisation analysis allowing for multiple causal variants. *PLoS Genet.* **17**, e1009440 (2021). 34587156 10.1371/journal.pgen.1009440 PMC8504726

[28] Giambartolomei, C. et al. Bayesian test for colocalisation between pairs of genetic association studies using summary statistics. *PLoS Genet.* **10**, e1004383 (2014). 24830394 10.1371/journal.pgen.1004383 PMC4022491

[29] Hormozdiari, F. et al. Colocalization of GWAS and eQTL signals detects target genes. *Am. J. Hum. Genet.* **99**, 1245–1260 (2016). 27866706 10.1016/j.ajhg.2016.10.003 PMC5142122

[30] Wen, X., Pique-Regi, R. & Luca, F. Integrating molecular QTL data into genome-wide genetic association analysis: probabilistic assessment of enrichment and colocalization. *PLoS Genet.* **13**, e1006646 (2017). 28278150 10.1371/journal.pgen.1006646 PMC5363995

[31] Urbut, S. M., Wang, G., Carbonetto, P. & Stephens, M. Flexible statistical methods for estimating and testing effects in genomic studies with multiple conditions. *Nat. Genet.* **51**, 187–195 (2019). 30478440 10.1038/s41588-018-0268-8 PMC6309609

[32] Kanai, M. et al. Meta-analysis fine-mapping is often miscalibrated at single-variant resolution. *Cell Genom.* **2**, 100210 (2022). 36643910 10.1016/j.xgen.2022.100210 PMC9839193

[33] Pasaniuc, B. & Price, A. L. Dissecting the genetics of complex traits using summary association statistics. *Nat. Rev. Genet.* **18**, 117–127 (2017). 27840428 10.1038/nrg.2016.142 PMC5449190

[34] Stephens, M. False discovery rates: a new deal. *Biostatistics* **18**, 275–294 (2017). 27756721 10.1093/biostatistics/kxw041 PMC5379932

[35] Zhao, Z., Banterle, M., Lewin, A. & Zucknick, M. Multivariate Bayesian structured variable selection for pharmacogenomic studies. *J. R. Stat. Soc. C* **73**, 420–443 (2024).

[36] Bottolo, L. et al. A computationally efficient Bayesian seemingly unrelated regressions model for high-dimensional quantitative trait loci discovery. *J. R. Stat. Soc. C* **70**, 886–908 (2021). 10.1111/rssc.12490 PMC7612194 35001978

[37] Bycroft, C. et al. The UK Biobank resource with deep phenotyping and genomic data. *Nature* **562**, 203–209 (2018). 30305743 10.1038/s41586-018-0579-z PMC6786975

[38] Astle, W. J. et al. The allelic landscape of human blood cell trait variation and links to common complex disease. *Cell* **167**, 1415–1429.e19 (2016). 27863252 10.1016/j.cell.2016.10.042 PMC5300907

[39] Kachuri, L. et al. Genetic determinants of blood-cell traits influence susceptibility to childhood acute lymphoblastic leukemia. *Am. J. Hum. Genet.* **108**, 1823–1835 (2021). 34469753 10.1016/j.ajhg.2021.08.004 PMC8546033

[40] Ulirsch, J. C. et al. Interrogation of human hematopoiesis at single-cell and single-variant resolution. *Nat. Genet.* **51**, 683–693 (2019). 30858613 10.1038/s41588-019-0362-6 PMC6441389

[41] Vuckovic, D. et al. The polygenic and monogenic basis of blood traits and diseases. *Cell* **182**, 1214–1231.e11 (2020). 32888494 10.1016/j.cell.2020.08.008 PMC7482360

[42] Chang, C. C. et al. Second-generation PLINK: rising to the challenge of larger and richer datasets. *Gigascience* **4**, 7 (2015). 25722852 10.1186/s13742-015-0047-8 PMC4342193

[43] Tokuhiro, S. et al. An intronic SNP in a RUNX1 binding site of SLC22A4, encoding an organic cation transporter, is associated with rheumatoid arthritis. *Nat. Genet.* **35**, 341–348 (2003). 14608356 10.1038/ng1267

[44] Scheitz, C. J. F. & Tumbar, T. New insights into the role of Runx1 in epithelial stem cell biology and pathology. *J. Cell. Biochem.* **114**, 985–993 (2013). 23150456 10.1002/jcb.24453 PMC5788165

[45] Asano, K. et al. Adult-onset eosinophilic airway diseases. *Allergy* **75**, 3087–3099 (2020). 33040364 10.1111/all.14620

[46] Helms, C. et al. A putative RUNX1 binding site variant between SLC9A3R1 and NAT9 is associated with susceptibility to psoriasis. *Nat. Genet.* **35**, 349–356 (2003). 14608357 10.1038/ng1268

[47] Gottlieb, P. A. (ed.) *Piezo Channels* Vol. 79, 97–134 (Academic Press, 2017).

[48] Cahalan, S. M. et al. Piezo1 links mechanical forces to red blood cell volume. *eLife* **4**, e07370 (2015). 26001274 10.7554/eLife.07370 PMC4456639

[49] Ling, T. & Crispino, J. D. GATA1 mutations in red cell disorders. *IUBMB Life* **72**, 106–118 (2020). 31652397 10.1002/iub.2177 PMC7323890

[50] Ma, S. et al. A role of PIEZO1 in iron metabolism in mice and humans. *Cell* **184**, 969–982.e13 (2021). 33571427 10.1016/j.cell.2021.01.024 PMC7927959

[51] Nichols, K. E. et al. Familial dyserythropoietic anaemia and thrombocytopenia due to an inherited mutation in GATA1. *Nat. Genet.* **24**, 266–270 (2000). 10700180 10.1038/73480 PMC10576470

[52] Han, B. & Eskin, E. Random-effects model aimed at discovering associations in meta-analysis of genome-wide association studies. *Am. J. Hum. Genet.* **88**, 586–598 (2011). 21565292 10.1016/j.ajhg.2011.04.014 PMC3146723

[53] Bovy, J., Hogg, D. W. & Roweis, S. T. Extreme deconvolution: inferring complete distribution functions from noisy, heterogeneous and incomplete observations. *Ann. Appl. Stat.* **5**, 1657–1677 (2011).

[54] Dawid, A. P. Some matrix-variate distribution theory: notational considerations and a Bayesian application. *Biometrika* **68**, 265–274 (1981).

[55] Gupta, A. K. & Nagar, D. K. *Matrix Variate Distributions* (Chapman & Hall, 2000).

[56] George, E. I. & McCulloch, R. E. Approaches for Bayesian variable selection. *Stat. Sin.* **7**, 339–373 (1997).

[57] Schwartzentruber, J. et al. Genome-wide meta-analysis, fine-mapping and integrative prioritization implicate new Alzheimer’s disease risk genes. *Nat. Genet.* **53**, 392–402 (2021). 33589840 10.1038/s41588-020-00776-w PMC7610386

[58] Sudlow, C. et al. UK Biobank: an open access resource for identifying the causes of a wide range of complex diseases of middle and old age. *PLoS Med.* **12**, e1001779 (2015). 25826379 10.1371/journal.pmed.1001779 PMC4380465

[59] Sheard, S. M., Nicholls, R. & Froggatt, J. *UK Biobank Haematology Data Companion Document* (UK Biobank, 2017).

[60] Weissbrod, O. et al. Functionally informed fine-mapping and polygenic localization of complex trait heritability. *Nat. Genet.* **52**, 1355–1363 (2020). 33199916 10.1038/s41588-020-00735-5 PMC7710571

[61] Mbatchou, J. et al. Computationally efficient whole-genome regression for quantitative and binary traits. *Nat. Genet.* **53**, 1097–1103 (2021). 34017140 10.1038/s41588-021-00870-7

[62] Horton, R. et al. Gene map of the extended human MHC. *Nat. Rev. Genet.* **5**, 889–899 (2004). 15573121 10.1038/nrg1489

[63] Caliskan, M., Brown, C. D. & Maranville, J. C. A catalog of GWAS fine-mapping efforts in autoimmune disease. *Am. J. Hum. Genet.* **108**, 549–563 (2021). 33798443 10.1016/j.ajhg.2021.03.009 PMC8059376

[64] Matzaraki, V., Kumar, V., Wijmenga, C. & Zhernakova, A. The MHC locus and genetic susceptibility to autoimmune and infectious diseases. *Genome Biol.* **18**, 76 (2017). 28449694 10.1186/s13059-017-1207-1 PMC5406920

[65] Raychaudhuri, S. et al. Five amino acids in three HLA proteins explain most of the association between MHC and seropositive rheumatoid arthritis. *Nat. Genet.* **44**, 291–296 (2012). 22286218 10.1038/ng.1076 PMC3288335

[66] Benner, C. et al. Prospects of fine-mapping trait-associated genomic regions by using summary statistics from genome-wide association studies. *Am. J. Hum. Genet.* **101**, 539–551 (2017). 28942963 10.1016/j.ajhg.2017.08.012 PMC5630179

[67] Schmidt, E. M. et al. GREGOR: evaluating global enrichment of trait-associated variants in epigenomic features using a systematic, data-driven approach. *Bioinformatics* **31**, 2601–2606 (2015). 25886982 10.1093/bioinformatics/btv201 PMC4612390

[68] Finucane, H. K. et al. Partitioning heritability by functional annotation using genome-wide association summary statistics. *Nat. Genet.* **47**, 1228–1235 (2015). 26414678 10.1038/ng.3404 PMC4626285

[69] Gusev, A. et al. Partitioning heritability of regulatory and cell-type-specific variants across 11 common diseases. *Am. J. Hum. Genet.* **95**, 535–552 (2014). 25439723 10.1016/j.ajhg.2014.10.004 PMC4225595

[70] Hormozdiari, F. et al. Leveraging molecular quantitative trait loci to understand the genetic architecture of diseases and complex traits. *Nat. Genet.* **50**, 1041–1047 (2018). 29942083 10.1038/s41588-018-0148-2 PMC6030458

[71] Vasquez, Y. M. et al. FOXO1 is required for binding of PR on IRF4, novel transcriptional regulator of endometrial stromal decidualization. *Mol. Endocrinol.* **29**, 421–433 (2015). 25584414 10.1210/me.2014-1292 PMC4347287

[72] Zou, Y., Carbonetto, P., Xie, D., Wang, G. & Stephens, M. PLINK association test statistics of UK Biobank blood traits (1.0). *Zenodo*10.5281/zenodo.8088040 (2023).

[73] Wang, G., Zou, Y., Carbonetto, P. & Stephens, M. stephenslab/mvsusieR: mvsusieR 0.1.8. *Zenodo*10.5281/zenodo.17296669 (2025).

[74] Zou, Y. & Carbonetto, P. stephenslab/finemap-uk-biobank: finemap-uk-biobank 1.0 (v1.0). *Zenodo*10.5281/zenodo.8400278 (2023).

[75] Zou, Y., Stephens, M. & Carbonetto, P. stephenslab/mmbr-rss-dsc: release of repository to accompany journal resubmission. (v1.1). *Zenodo*10.5281/zenodo.8087907 (2024).

[76] gaow et al. gaow/mvarbvs: add mvSuSiE prior and analysis pipelines (v0.9.2). *Zenodo*10.5281/zenodo.8094982 (2024).

