# Supplementary material for: Fast and flexible joint fine-mapping of multiple traits via the Sum of Single Effects model
Source: Nat Genet. 2026 Feb 3;58(2):454–62. doi: 10.1038/s41588-025-02486-7 (PMC12900646; doi:10.1038/s41588-025-02486-7)
Supplement: Supplementary file 3 — Peer Review File [file 41588_2025_2486_MOESM3_ESM.pdf]

nature portfolio

Peer Review File

Fast and flexible joint fine-mapping of multiple traits via the Sum of Single Effects model

Corresponding Author: Dr Matthew Stephens

Version 0:

Decision Letter:

1st May 2023

Dear Dr Stephens,

Thank you for submitting your manuscript entitled "Fast and flexible joint fine-mapping of multiple traits via the Sum of Single Effects model". We have given the paper our careful consideration and find it of potential interest. However, we will need to receive necessary forms before sending your manuscript for peer review.

The Reporting Summary and Editorial Policy Checklist can be found using the links below:

-- Reporting summary: https://www.nature.com/documents/nr-reporting-summary.pdf

-- Editorial policy checklist: https://www.nature.com/documents/nr-editorial-policy-checklist.pdf

-- Code availability: Please refer to “Nature Research_Code Guidelines.pdf” for details. Code will need to be made available to reviewers for testing.

Please complete the forms as soon as possible. Please note that the forms are dynamic ‘smart pdfs’ and must therefore be downloaded and completed in Adobe Reader. We will then flatten it for ease of use by the reviewers. If you would like to reference the guidance text as you complete the template, please access these flattened versions at http://www.nature.com/authors/policies/availability.html.

Note that you are not required to revise your paper to include the information provided in the reporting summary. However, all points on the policy checklist must be addressed; please send me a new version of the manuscript with your completed checklist if needed.

Please be aware of our guidelines on digital image standards.

Please use the link below to upload the manuscript files and necessary forms.

Link Redacted

Nature Genetics is committed to improving transparency in authorship. As part of our efforts in this direction, we are now requesting that all authors identified as ‘corresponding author’ on published papers create and link their Open Researcher and Contributor Identifier (ORCID) with their account on the Manuscript Tracking System (MTS), prior to acceptance. ORCID helps the scientific community achieve unambiguous attribution of all scholarly contributions. You can create and link your ORCID from the home page of the MTS by clicking on ‘Modify my Springer Nature account’. For more information please visit please visit www.springernature.com/orcid.

Thank you for your interest in Nature Genetics.

Sincerely,

Wei Li, PhD
Senior Editor

Nature Genetics
New York, NY 10004, USA
www.nature.com/ng

Version 1:

Decision Letter:

10th Aug 2023

Dear Dr Stephens,

Your Technical Report, "Fast and flexible joint fine-mapping of multiple traits via the Sum of Single Effects model" has now been seen by 3 referees. You will see from their comments below that while they find your work of interest, some important points are raised. We are interested in the possibility of publishing your study in Nature Genetics, but would like to consider your response to these concerns in the form of a revised manuscript before we make a final decision on publication.

To guide the scope of the revisions, the editors discuss the referee reports in detail within the team with a view to identifying key priorities that should be addressed in revision. In this case, we think all three referees have provided constructive reviews aimed at strengthening the analyses and improving the presentation, and we particularly ask that you clarify the limitations of mvSuSiE, improve the real-data analysis, and address all referee comments as thoroughly as possible with appropriate revisions. We hope that you will find the prioritized set of referee points to be useful when revising your study.

We therefore invite you to revise your manuscript taking into account all reviewer and editor comments. Please highlight all changes in the manuscript text file. At this stage we will need you to upload a copy of the manuscript in MS Word .docx or similar editable format.

We are committed to providing a fair and constructive peer-review process. Do not hesitate to contact us if there are specific requests from the reviewers that you believe are technically impossible or unlikely to yield a meaningful outcome.

When revising your manuscript:

*1) Include a “Response to referees” document detailing, point-by-point, how you addressed each referee comment. If no action was taken to address a point, you must provide a compelling argument. This response will be sent back to the referees along with the revised manuscript.

*2) If you have not done so already please begin to revise your manuscript so that it conforms to our Technical Report format instructions, available
here.
Refer also to any guidelines provided in this letter.

*3) Include a revised version of any required Reporting Summary: https://www.nature.com/documents/nr-reporting-summary.pdf
It will be available to referees (and, potentially, statisticians) to aid in their evaluation if the manuscript goes back for peer review.
A revised checklist is essential for re-review of the paper.

Please be aware of our guidelines on digital image standards.

Please use the link below to submit your revised manuscript and related files:

Link Redacted

Note: This URL links to your confidential home page and associated information about manuscripts you may have submitted, or that you are reviewing for us. If you wish to forward this email to co-authors, please delete the link to your homepage.

We hope to receive your revised manuscript within 3 to 6 months. If you cannot send it within this time, please let us know.

Please do not hesitate to contact me if you have any questions or would like to discuss these revisions further.

Nature Genetics is committed to improving transparency in authorship. As part of our efforts in this direction, we are now requesting that all authors identified as ‘corresponding author’ on published papers create and link their Open Researcher and Contributor Identifier (ORCID) with their account on the Manuscript Tracking System (MTS), prior to acceptance. ORCID helps the scientific community achieve unambiguous attribution of all scholarly contributions. You can create and link your ORCID from the home page of the MTS by clicking on ‘Modify my Springer Nature account’. For more information please visit please visit www.springernature.com/orcid.

We look forward to seeing the revised manuscript and thank you for the opportunity to review your work.

Sincerely,
Wei

Wei Li, PhD
Senior Editor
Nature Genetics
New York, NY 10004, USA
www.nature.com/ng

Reviewers' Comments:

Reviewer #1:
Remarks to the Author:
In this manuscript, Zou et al developed and evaluated a novel multi-trait fine-mapping method, mvSuSiE. This method was an extension of a published and well-received single-trait fine-mapping method, SuSiE, which was also developed by the authors' team. I find mvSuSiE solid, thoroughly evaluated, and timely given the increasing global biobank efforts. I especially like the thoughts and novelty in summarizing the inference across traits. The manuscript was well written. I have a few comments for the authors to consider:

Major:
1. Throughout the manuscript, the authors used an empirical threshold of lfsr < 0.01. How was it determined? The authors claimed to find genetic associations with many blood traits in the EST1-SAMD12 locus that the previous GWAS failed to find (Figure 4A). But the finding was really based on this 0.01 threshold that was not justified (while GWAS used the 5E-8 "Bonferroni" threshold). In a sense this is a bit unfair. Could the authors set this threshold using a principled approach?
2. The authors mentioned a limitation that mvSuSiE requires traits to be measured on all samples. The authors also mentioned "a general approach to deal with missing data" could potentially alleviate this limitation. I wonder if the authors could comment a bit more in this space. This limitation will preclude a lot of applications for mvSuSiE. For example, many disease genetic studies share the same control but have individuals diagnosed with different but related disorders that can be mutually exclusive (e.g., schizophrenia and bipolar). Do the authors have any recommendations for such studies? If mvSiSiE shouldn't be used for these studies, perhaps the authors should make it clear to prevent misuse.
3. The correlation structure (U) was learned genome-wide from top SNPs. However, effect size correlation can sometimes be local (e.g., as discussed in several local genetic correlation papers). I wonder if the authors would comment on the implication.
4. I wonder if the authors could comment on applying mvSuSiE to case control studies. And what if the summary statistics are generated from a linear mixed model such as Regenie rather than the linear model as used in the UKBB example (as LMM has gained much popularity with biobank studies)?

Minor
5. Lines 436-441: it'd be helpful to give a rationale and intuition for clfsr.
6. Line 146-148: I think the authors meant the "random effects prior" when saying "forced mvSuSiE to make the same independence assumptions as CAFEH". However, Supplementary Figures 1-2 don't fully support what the authors said in the text: "random effects prior" still appear to outperform CAFEH in scenario b, e.g., in the coverage and median size.
7. Didn't "1-SNP CS" and "median of purity" panels in Figure 2c show that mvSuSiE is doing worse than CAFEH?
8. Figure 3 legend needs more details, e.g., how was the proportion of CS in panel a calculated?
9. It can be helpful to add the grouping of blood cells in figure 4, e.g., what are the cell types that are "mature red blood cells". I understand the authors might have included this info, but it'd be good to show it with the figure to make it easier for the readers.
10. Perhaps the authors can do a better job connecting Figure 1 to the method section. For example, the authors could make it clear that panel c is related to the approach for estimating V (Eq 24) and e for estimating U (cite the discussions in the prior)
11. Is I_R in the canonical prior the same as "Random effects prior"? If so perhaps the authors should remove the random effects prior (lines 485-488) and use only the remaining two categories of priors (canonical and data-driven) to avoid confusion.
12. Supplementary Figure 5: why well-calibrated methods should have points on the diagonal line? I understand if it's under null for a QQ plot but I thought this is something different.
13. Supplementary Figures 6-10: could the authors label each panel for its underlying covariance matrix (e.g, equal effect, rank -3 and etc)?

Reviewer #2:
Remarks to the Author:
Review: Fast and flexible joint fine-mapping of multiple traits via the Sum of Single Effects model
Overview: Zou et al present mvSuSiE, a multivariate extension of the ultra-scalable "Sum of Single Effects" fine-mapping

model that seeks to jointly fine-map multiple traits/outcomes. While there exists methods for fine-mapping multiple traits jointly, they either fail to scale to large datasets, or do not leverage a shared architecture assumption, which may lose power when it exists. mvSuSiE addresses these limitations by incorporating the SuSiE model for fast inference, while using multivariate priors on effect-sizes across traits that allow for correlations to exist a-priori. The authors validate their approach in simulations and analyze large-scale GWAS of blood traits in the UK Biobank. I found the manuscript well written and timely. With that said, I have some comments.

Major Comments:
1. The authors performed extensive simulations that were informed from real data scenarios. While this helps understand behavior in typical use cases (which is important), I found it hard to parse with respect to specific modeling aspects.

It would be helpful to see how mvSuSiE performs (or outperforms) as residual correlations increase, or similarly as correlations in effect sizes increase. This would provide helpful information to see the extent of performance along a gradient of scales.

Similarly, how much of the improvement of mvSuSie comes from refinement over mvIBSS results?

2. I was excited to see the real-data results and their interpretations but found myself underwhelmed with the results as reported. The authors describe the improvement in credible sets identified and single-SNP credible sets, but it is less clear if the method is discovering novel biological signal, or if it is a consequence of false positives on average. The examples listed certainly help, but a more broad analysis would place the general results in a clearer light.

One possible way to dissect this (albeit with its own flaws) would be to perform functional enrichment using trait/cell matched annotations. Are cell/trait matched functional annotations more enriched for PIPs/CS signals from mvSuSiE than those inferred from SuSiE?

3. Similarly, the authors focus on comparisons between CAFE and SuSiE in simulations, but only compare with SuSiE in real data analysis. While comparison against SuSiE is helpful to see gains due to multivariate assumptions, it would also help to assess gains due improved modeling assumptions in mvSuSiE vs CAFE (e.g., non-independence of traits).

Minor Comments:
1. No color legend is supplied for Fig 3E.

Reviewer #3:
Remarks to the Author:
The authors extend SUSIE for single-trait fine-mapping to multiple traits, mvSUSIE. It can be used with individual-level or summary data, but requires all traits to be measured on each individual in the cohort (i.e. no missing trait measurements). Simulations show that 20 traits can be jointly fine-mapped, and they use mvSUSIE to jointly finemap signals from 16 blood cell traits in UK Biobank. Some detailed comments follow.

Major
1. Line 116-117 – the simulation comparison between mvSUSIE, PAINTOR, and flashfm is incomplete. A more realistic scenario with two correlated traits (rather than independent) that share at least one causal variant, though not all shared, is needed. Also, credible set sizes should be compared for all methods.
2. Lines 139-140 Proportion of 1-SNP credible sets is given for mvSUSIE and CAFEH comparisons, but it is also important to consider the size of these sets when there is more than one SNP in the set. Please include a comparison of credible set sizes for these methods.
3. A discussion is needed on the trade-off between joint analysis of many traits in a reduced sample size compared to a joint analysis of fewer traits in a larger sample or even a single trait in a larger sample. Their UK Biobank analysis had n~ 250k, where all 16 traits were measured in the individuals, but the previous analysis by Vuckovic et al. had n ~ 408k. It would be helpful to include an application of SUSIE to the complete UK Biobank data for some of the highlighted examples.

Minor
1. Line 25 – cite which methods are limited to fine-mapping only two traits
2. Line 25-25 – cite which methods are limited by 1-2 causal variants
3. Table 1 – add a column for upper limit on the number of traits for each method
4. Line 59-67 – the other multi-trait methods (PAINTOR, flashfm) that are in the simulations should be discussed here.
5. Line 153 – please provide the details of the set of simulations that compare mvSUSIE with PAINTOR and flashfm – how many SNPs in the region, how many causal SNPs, how many shared causal SNPs?
6. Supp Fig 4 – it is difficult to see the details for all methods, so please list the median (and quartiles) running times for all three methods.
7. Lines 707-709 – This statement is confusing and could cause errors when running flashfm. Users will get incorrect results if they input a 0-vector for the effect allele frequencies (EAF) to any of the flashfm wrapper functions, or to the flashfm function if the trait means are non-zero.

Version 2:

Decision Letter:

15th Apr 2024

Dear Professor Stephens,

Your Technical Report, "Fast and flexible joint fine-mapping of multiple traits via the Sum of Single Effects model" has now been seen by 3 referees. You will see from their comments below that while they find your work of interest, some important points are raised. We are interested in the possibility of publishing your study in Nature Genetics, but would like to consider your response to these concerns in the form of a revised manuscript before we make a final decision on publication.

As you will see from the reviewer comments, we think Reviewers 1 & 3 have identified important aspects of the study design and the analyses that need to be substantially improved. We particularly ask that you address their technical comments as thoroughly as possible with appropriate revisions.

We therefore invite you to revise your manuscript taking into account all reviewer and editor comments. Please highlight all changes in the manuscript text file. At this stage we will need you to upload a copy of the manuscript in MS Word .docx or similar editable format.

We are committed to providing a fair and constructive peer-review process. Do not hesitate to contact us if there are specific requests from the reviewers that you believe are technically impossible or unlikely to yield a meaningful outcome.

When revising your manuscript:

*1) Include a “Response to referees” document detailing, point-by-point, how you addressed each referee comment. If no action was taken to address a point, you must provide a compelling argument. This response will be sent back to the referees along with the revised manuscript.

*2) If you have not done so already please begin to revise your manuscript so that it conforms to our Technical Report format instructions, available
here.
Refer also to any guidelines provided in this letter.

*3) Include a revised version of any required Reporting Summary: https://www.nature.com/documents/nr-reporting-summary.pdf
It will be available to referees (and, potentially, statisticians) to aid in their evaluation if the manuscript goes back for peer review.
A revised checklist is essential for re-review of the paper.

Please be aware of our guidelines on digital image standards.

Please use the link below to submit your revised manuscript and related files:

Link Redacted

Note: This URL links to your confidential home page and associated information about manuscripts you may have submitted, or that you are reviewing for us. If you wish to forward this email to co-authors, please delete the link to your homepage.

We hope to receive your revised manuscript within four to eight weeks. If you cannot send it within this time, please let us know.

Please do not hesitate to contact me if you have any questions or would like to discuss these revisions further.

Nature Genetics is committed to improving transparency in authorship. As part of our efforts in this direction, we are now requesting that all authors identified as ‘corresponding author’ on published papers create and link their Open Researcher and Contributor Identifier (ORCID) with their account on the Manuscript Tracking System (MTS), prior to acceptance. ORCID helps the scientific community achieve unambiguous attribution of all scholarly contributions. You can create and link your ORCID from the home page of the MTS by clicking on ‘Modify my Springer Nature account’. For more information please visit please visit www.springernature.com/orcid.

We look forward to seeing the revised manuscript and thank you for the opportunity to review your work.

Sincerely,
Wei

Wei Li, PhD

Senior Editor
Nature Genetics
New York, NY 10004, USA
www.nature.com/ng

Reviewers' Comments:

Reviewer #1:
Remarks to the Author:
The authors have addressed most of my concerns. mvSuSiE is a novel, solid, and very timely fine-mapping method. My remaining questions mainly focus on improving the interpretation of the results (follow up of the previous comment 1) and the method's utility (follow up of the previous comment 2).

Interpretation of results (follow up of comment #1):

I agree with the authors that a choice of threshold is arbitrary, and mvSuSiE is a Bayesian method that estimates the probability, which differs from the frequentist approach. However, I found the interpretation of lfsr unclear, which prevented its mvSuSie's utility as a Bayesian method. For example, the authors said, "lfsr … are estimates of an error rate" in the response; what is the error lfsr measures? The manuscript defines lfsr as "how confident we can be in the sign of the effect." How does lfsr quantify "confidence"? For example, can lfsr be interpreted as the probability that the CS is not causal to trait r? The author brought up FDR and P-value in the response, I think giving lfsr a clear definition as FDR and P-value would be very helpful.

Regarding the threshold, indeed, any threshold is arbitrary. However, future studies using mvSuSiE will likely use the 0.01 threshold the author used in the manuscript. I'd like to see this threshold can be justified as reasonable for future studies.

Utility (follow up of comment #2):

The authors mentioned in Discussions that "The multivariate linear model (1) assumes that the traits are measured in the same samples". How bad if this assumption is violated? Summary statistics from consortia (e.g., Psychiatric Genomics Consortium) can have highly related traits generated on datasets from different samples, often with overlaps. Clarifying this can make sure mvSuSiE is used appropriately in such cases.

Reviewer #2:
Remarks to the Author:
The authors have addressed my original comments. I have no new comments at this time.

Reviewer #3:
Remarks to the Author:
The authors have not addressed all comments, and vague and misleading answers have been provided. All simulation comparisons need more explanations/details.

1. The simulation comparison of mvSuSiE with PAINTOR and flashfm is still incomplete. Although the authors now include comparisons where the traits are correlated, they have not used a realistic setting where there are multiple causal variants for each trait and not all causal variants are shared between the traits (as suggested). The two-trait simulation is still for only causal variants shared by both traits.

2. Are the cross-trait PP not used at all for trait-specific mvSusie results? I was under the impression that trait-specific variants should have high cross-trait PP (so causal for at least one trait) with small lfsr for the particular trait, but this does not seem to be the case in the trait-specific simulations. The authors must explain how to identify causal SNPs and which traits are affected by them and then, evaluate this approach in their trait-wise simulation comparisons with CAFEH and flashfm.

3. The trait-specific power-FDR plots are misleading because they vary thresholds for two very different measures, PIP/MPP (for CAFEH, Susie, flashfm) and lfsr (for mvSusie). The only disclosed thresholds are at baseline (open circles) with a more lenient lfsr threshold of 0.05 compared to stringent PIP/MPP thresholds of 0.95; in the data application, the authors used lfsr threshold 0.01 and PIP threshold 0.95, and they should display both in simulation comparisons. Also, as detailed above, it needs to be clarified whether thresholds for cross-trait PP and lfsr are used together in mvSusie for trait-wise results – this does not seem to be the case in the simulations.

4. Figure legends lack details and they should contain enough information to understand the comparisons made in each figure. It is not clear how each plot is produced, power definitions, specific thresholds for each method, etc. The narrative of

the paper and key takeaways are based on such pictorial representations.

5. There are no convincing arguments of why average lfsr of 0.05 is used in some simulation comparisons instead of lfsr for each causal variant for a particular trait – the 0.05 threshold is also questionable. I see in the UK Biobank application that lfsr<0.01 was used to determine the significant CSs.

6. Supp Figs 5, 6, 7, 8 – median CS size is just a point estimate and not very informative without any bounds or indication of the distribution. Such bespoke choices require reasonable pieces of evidence to become good practice.

Version 3:

Decision Letter:

14th Aug 2024

Dear Professor Stephens,

Your Technical Report entitled "Fast and flexible joint fine-mapping of multiple traits via the Sum of Single Effects model" has now been seen by 2 referees, whose comments are attached. In the light of their advice we have decided that we cannot offer to publish your manuscript in Nature Genetics.

While the referees find your work of some interest, they raise serious concerns about the inconsistencies in threshold selection, the performance of this method and the technical advance over earlier work. We feel that these reservations are sufficiently important as to preclude publication of this study in Nature Genetics.

I am sorry that we cannot be more positive on this occasion but hope that you will find our referees' comments helpful when preparing your paper for submission elsewhere.

Sincerely,
Wei

Wei Li, PhD
Senior Editor
Nature Genetics
www.nature.com/ng

Reviewers' Comments:

Reviewer #1:
Remarks to the Author:
The authors have addressed all my concerns in this revision. I have no further comments.

Reviewer #3:
Remarks to the Author:
Major issues remain from the previous revision.

1. There are even more inconsistencies with threshold choices for different metrics than before. Previously, I recommended the baseline comparison as lfsr=0.01 vs PIP/MPP=0.95 in their simulations, since these thresholds are used in their data application. This has not been done, though they agreed that it should be done. They now correctly include lfsr threshold 0.01, but have oddly increased the PIP threshold to an extraordinary threshold of 0.99, and imply in various places that lfsr=1-PIP is a comparable threshold to PIP (e.g. Fig 2b, Supp Table 1). There is no factual reasoning in using an extraordinary PIP/MPP threshold 0.99 (especially when 95% credible sets are the focus), except to attain obfuscating comparisons. In fact, it would have been more useful to include sensible PIP/MPP thresholds (like 0.8 to 0.9, being more lenient) as they have included a more lenient lfsr=0.05 threshold. It is factually (extremely) misleading to imply that lfsr=0.01 (their recommended high-confidence threshold) is comparable to PIP=0.99, as in their data analysis they have worked under the assumption that lfsr=0.01 and PIP=0.95 are comparable.

Supp Fig 3 still uses lfsr=0.05 instead of 0.01. Supp Fig 5 uses lfsr=0.01, but oddly increases the PIP/MPP threshold to 0.99, which is more stringent and never used in methods comparisons or in practice. Supp fig 5a should be similar to Supp Fig 3b (for methods appearing in both), and the threshold discrepancies are clear. PIP/MPP thresholds in the remaining supp figures are not stated, so unclear if 0.95 is used, or if there has been an incorrect increase to 0.99 there too.

2. There are still numerous inconsistencies in the use of their stage 1 cross-trait PIP together with stage 2 lfsr in their cross-trait simulation results. They confirmed, as I understood previously, that these two steps are used in their UK Biobank analysis, but they do not address why they do not replicate these steps in their simulations. Simulations are used to understand how the methods perform when we know the truth, so it is customary to use the same approach in the data analysis as in the simulations, where the simulations give evidence for the proposed approach.

3. Many thanks for trying to explain the power vs FDR plots, but I am very much aware of this. However, in the context of fine-mapping these are not practical. When fine-mapping, it is common for the analyst to pre-specify a PIP threshold to decide on which variants pass as high-confidence. Therefore, it must be known what PIP/MPP/lfsr threshold is used at (FDR, power) points because when applying these fine-mapping methods, those are the thresholds that will be pre-specified, not FDR. The authors must indicate which threshold levels were used at various points, so that users are able to understand the implications of various threshold choices and any advantages to using this method.

**Although we cannot publish your paper, it may be appropriate for another journal in the Nature Portfolio. If you wish to explore the journals and transfer your manuscript please use our manuscript transfer portal. You will not have to re-supply manuscript metadata and files, unless you wish to make modifications. For more information, please see our manuscript transfer FAQ page.

Version 4:

Decision Letter:

IMPORTANT: Please note the reference number: NG-TR62293R3-Z Stephens. This number must be quoted whenever you communicate with us regarding this paper.

8th Nov 2024

Dear Dr. Stephens,

Thank you for your message of asking us to reconsider our decision on your manuscript "Fast and flexible joint fine-mapping of multiple traits via the Sum of Single Effects model". I have now discussed the points of your letter with my colleagues, and we think that you have some valid points. We therefore invite you to upload your revised manuscript and updated forms (Editorial Policy Checklist & Reporting Summary) for peer review.

When preparing a revision, please ensure that it fully complies with our editorial requirements for format and style; details can be found in the Guide to Authors on our website (http://www.nature.com/ng/).

Please be sure that your manuscript is accompanied by a separate letter detailing the changes you have made and your response to the points raised. At this stage we will need you to upload:
1) a copy of the manuscript in MS Word .docx format.
2) A point-by-point response to reviewer(s)
3) an updated Editorial Policy Checklist:
https://www.nature.com/documents/nr-editorial-policy-checklist.pdf
4) an updated Reporting Summary:
https://www.nature.com/documents/nr-reporting-summary.pdf
(Here you can read about the role of the Reporting Summary in reproducible science:
https://www.nature.com/news/announcement-towards-greater-reproducibility-for-life-sciences-research-in-nature-1.22062)

Please use the link below to be taken directly to the site and view and revise your manuscript:

Link Redacted

With kind wishes,

Wei Li, PhD
Senior Editor
Nature Genetics
www.nature.com/ng

Version 5:

Decision Letter:

11th Jun 2025

Dear Dr Stephens,

Your Technical Report, "Fast and flexible joint fine-mapping of multiple traits via the Sum of Single Effects model" has now been seen by 3 referees. You will see from their comments below that while they find your work of interest, some important points are raised. We are interested in the possibility of publishing your study in Nature Genetics, but would like to consider your response to these concerns in the form of a revised manuscript before we make a final decision on publication.

We therefore invite you to revise your manuscript taking into account all reviewer and editor comments. Please highlight all changes in the manuscript text file. At this stage we will need you to upload a copy of the manuscript in MS Word .docx or similar editable format.

We are committed to providing a fair and constructive peer-review process. Do not hesitate to contact us if there are specific requests from the reviewers that you believe are technically impossible or unlikely to yield a meaningful outcome.

When revising your manuscript:

*1) Include a “Response to referees” document detailing, point-by-point, how you addressed each referee comment. If no action was taken to address a point, you must provide a compelling argument. This response will be sent back to the referees along with the revised manuscript.

*2) If you have not done so already please begin to revise your manuscript so that it conforms to our Technical Report format instructions, available
here.
Refer also to any guidelines provided in this letter.

*3) Include a revised version of any required Reporting Summary: https://www.nature.com/documents/nr-reporting-summary.pdf
It will be available to referees (and, potentially, statisticians) to aid in their evaluation if the manuscript goes back for peer review.
A revised checklist is essential for re-review of the paper.

Please be aware of our guidelines on digital image standards.

EXTENDED DATA FIGURES

When re-submitting your manuscript, please ensure that any supplementary figures and tables that are crucial to the manuscript’s conclusions are converted into Extended Data figures and tables to increase visibility of these data. Extended Data figures and tables are online-only (present in the online PDF and full-text HTML versions of the paper), peer-reviewed display items that provide essential background to the article but are not included in the main article due to space constraints. A maximum of ten Extended Data display items (figures and tables) is permitted.

Please use the link below to submit your revised manuscript and related files:

Link Redacted

Note: This URL links to your confidential home page and associated information about manuscripts you may have submitted, or that you are reviewing for us. If you wish to forward this email to co-authors, please delete the link to your homepage.

We hope to receive your revised manuscript within four to eight weeks. If you cannot send it within this time, please let us know.

Please do not hesitate to contact me if you have any questions or would like to discuss these revisions further.

Nature Genetics is committed to improving transparency in authorship. As part of our efforts in this direction, we are now requesting that all authors identified as ‘corresponding author’ on published papers create and link their Open Researcher and Contributor Identifier (ORCID) with their account on the Manuscript Tracking System (MTS), prior to acceptance. ORCID helps the scientific community achieve unambiguous attribution of all scholarly contributions. You can create and link your ORCID from the home page of the MTS by clicking on ‘Modify my Springer Nature account’. For more information please visit please visit www.springernature.com/orcid.

We look forward to seeing the revised manuscript and thank you for the opportunity to review your work.

Sincerely,

Wei Li, PhD
Senior Editor
Nature Genetics
www.nature.com/ng

Reviewers' Comments:

Reviewer #1 (Remarks to the Author):

I believe the authors have addressed Reviewer 3’s comments 1 and 2 effectively. The mvSuSiE method has a complex design, incorporating two measures: PIP and lfsr. While PIP is widely used in fine-mapping studies, lfsr is relatively novel and not commonly employed in most existing methods. As the authors have noted, lfsr is fundamentally different from PIP, which makes direct comparisons with other methods difficult or even infeasible.

Further complicating such comparisons is the fact that PIP values are not calibrated across fine-mapping methods, as noted in several publications (e.g., https://pubmed.ncbi.nlm.nih.gov/36643910/). In this context, I find the use of FDR to be appropriate and justified, as it has been used to align results across different fine-mapping approaches fairly.

Overall, I think the authors have done a good job responding to Reviewer 3’s comments 1 and 3. The thresholds are now aligned, and the manuscript includes clear notes cautioning readers about the limitations in comparing methods. One optional suggestion I have is to add a brief explanation in the Discussion section about the use of lfsr, particularly for readers who may not be methodological experts. As I mentioned previously, my concern is the potential misuse or misinterpretation of lfsr as a novel measure.

Regarding comment 2, it appears the authors did use the “stage 2 lfsr” in the UK Biobank analysis, as indicated in Line 242: “The number of CSs significant in each trait (average lfsr < 0.01).” This seems to contradict the statement that “these steps are not used in the UK Biobank analyses.” It would be helpful for the authors to clarify this point.

Finally, I noticed a minor error in this version: in Equation 23 of the supplement, the summation should be over SNPs (indexed by J), not the number of effects (L).

Reviewer #2 (Remarks to the Author):

It has been some time since my initial review, but will add my remarks on the validity of points made by Reviewer #3 as well as the authors' response.

First, regarding the choice of thresholds and Power/FDR curves.

I find myself agreeing -to some extent- on the utility of Power/FDR curves in fine-mapping papers. While these curves have gained popularity in recent years, and are a staple in general statistical settings, I feel their inclusion has been somewhat detrimental to not only fine-mapping method developers but also consumers of these tools.

Namely, fine-mapping is a challenging variable selection problem, where our knowledge of the underlying biology has driven the development of statistical concepts to reflect this. For example, credible sets provide an interpretable (largely) means to capture putative causal variants after accounting for LD. Straightforward metrics in simulations based on sensitivity/calibration of PIPs and credible sets are helpful and maps back to how practitioners THINK AND USE these tools.

In contrast, Power/FDR curves may be overly conservative/unrealistic due to LD complications (ie single causal in perfect LD => PIP of .5 would penalize these curves, but be completely reasonable in a CS metric), notions of variable thresholds, and when practitioners decide to fine-map in the first place.

Now with that said, I have no strong concerns about their use in simulations to present some notion of performance as they are still informative, but with their own limitations. Similarly, I am sympathetic to the authors regarding minor typos or missed changes in supplement when a large number of supplemental materials exist, provided the authors make their best effort to correct/address this (which it seems they have).

The remaining concerns raised by Reviewer #3 seem to be a misunderstanding on their part, which is not due to a lack of information from the authors from what I can gather. Their approach was outlined in a previous submission which may have been missed by the reviewer.

Reviewer #3 (Remarks to the Author):

Transparency in results has been a consistent theme in my comments throughout the review process. From the early stages, I requested details on the thresholds used at various points in the plots to assess how the methods perform at typical analysis thresholds. Only in the fourth revision has the bare minimum level of transparency been met, with details now provided for two plausible and somewhat comparable thresholds. I understand the authors' frustration, but providing underlying data for plots is standard practice.

With this minimum level of transparency now in place, it becomes clear that the new approach offers no significant gains over existing multi-trait methods. The previous revision included two threshold points, but one was an unrealistically stringent PIP>0.99 for current methods — something not used in practice and now removed from this revision.

There is a discrepancy between implied outperformance and tangible evidence-based outperformance of the proposed method over the existing ones. The narrative of the paper is that the proposed method outperforms the existing methods, and the method comparison visualizations give the impression of even larger improvements. For example, the plots exaggerate differences by focusing on thresholds not used in practice. Supplementary tables show FDR is generally <0.05 at lenient thresholds (PIP>0.80, lfsr<0.05), reinforcing my concern about misleading interpretations from the power-FDR plots. At these thresholds, power differences are minimal — often only in the second digit — and visible only in the supplementary tables. These results are hard to discern in the plots due to the scale. As stated in previous reviews, users need to understand how methods perform at realistic thresholds.

While the authors have acknowledged inconsistencies and corrected some plots, issues remain. In my last review, I noted inconsistencies in Supp Figs 3 and 5 and stated: “PIP/MPP thresholds in the remaining supp figures are not stated, so unclear if 0.95 is used, or if there has been an incorrect increase to 0.99 there too.” This remains unresolved in Supp Figs 6, 7, and others. Given I already raised this in the previous revision, it is something the authors could have resolved.

Version 6:

Decision Letter:

Our ref: NG-TR62293R5

14th Aug 2025

Dear Dr. Stephens,

Thank you for submitting your revised manuscript "Fast and flexible joint fine-mapping of multiple traits via the Sum of Single Effects model" (NG-TR62293R5). The reviewers find that the paper has improved in revision, and therefore we'll be happy in principle to publish it in Nature Genetics, pending minor revisions to satisfy the referees' final requests and to comply with our editorial and formatting guidelines.

We are now performing detailed checks on your paper and will send you a checklist detailing our editorial and formatting requirements soon. Please do not upload the final materials and make any revisions until you receive this additional information from us.

Thank you again for your interest in Nature Genetics Please do not hesitate to contact me if you have any questions.

Sincerely,

Wei Li, PhD
Senior Editor
Nature Genetics
www.nature.com/ng

Version 7:

Decision Letter:

In reply please quote: NG-TR62293R6 Stephens

12th Dec 2025

Dear Dr. Stephens,

I am delighted to say that your manuscript "Fast and flexible joint fine-mapping of multiple traits via the Sum of Single Effects model" has been accepted for publication in an upcoming issue of Nature Genetics.

Over the next few weeks, your paper will be copyedited to ensure that it conforms to Nature Genetics style. Once your paper is typeset, you will receive an email with a link to choose the appropriate publishing options for your paper and our Author Services team will be in touch regarding any additional information that may be required.

After the grant of rights is completed, you will receive a link to your electronic proof via email with a request to make any corrections within 48 hours. If, when you receive your proof, you cannot meet this deadline, please inform us at rjsproduction@springernature.com immediately.

You will not receive your proofs until the publishing agreement has been received through our system.

Due to the importance of these deadlines, we ask that you please let us know now whether you will be difficult to contact over the next month. If this is the case, we ask you provide us with the contact information (email, phone and fax) of someone who will be able to check the proofs on your behalf, and who will be available to address any last-minute problems.

Your paper will be published online after we receive your corrections and will appear in print in the next available issue. You can find out your date of online publication by contacting the Nature Press Office (press@nature.com) after sending your e-proof corrections.

You may wish to make your media relations office aware of your accepted publication, in case they consider it appropriate to organize some internal or external publicity. Once your paper has been scheduled you will receive an email confirming the publication details. This is normally 3-4 working days in advance of publication. If you need additional notice of the date and time of publication, please let the production team know when you receive the proof of your article to ensure there is sufficient time to coordinate. Further information on our embargo policies can be found here: https://www.nature.com/authors/policies/embargo.html

Before your paper is published online, we shall be distributing a press release to news organizations worldwide, which may very well include details of your work. We are happy for your institution or funding agency to prepare its own press release, but it must mention the embargo date and Nature Genetics. Our Press Office may contact you closer to the time of publication, but if you or your Press Office have any enquiries in the meantime, please contact press@nature.com.

Acceptance is conditional on the data in the manuscript not being published elsewhere, or announced in the print or electronic media, until the embargo/publication date. These restrictions are not intended to deter you from presenting your data at academic meetings and conferences, but any enquiries from the media about papers not yet scheduled for publication should be referred to us.

Authors may need to take specific actions to achieve compliance with funder and institutional open access mandates. If your research is supported by a funder that requires immediate open access (e.g. according to Plan S principles or the NIH public access policy) then you should select the gold OA route, and we will direct you to the compliant route where possible. Because authors warrant under our subscription licensing terms that they haven't committed to licensing any version of their article under a licence inconsistent with the terms of our agreement – including the applicable embargo period – publication under the subscription model isn't suitable for authors whose funders require no embargo.

If you have any questions about our publishing options, costs, Open Access requirements, or our legal forms, please contact ASJournals@springernature.com

If you have posted a preprint on any preprint server, please ensure that the preprint details are updated with a publication reference, including the DOI and a URL to the published version of the article on the journal website.

To assist our authors in disseminating their research to the broader community, our SharedIt initiative provides you with a unique shareable link that will allow anyone (with or without a subscription) to read the published article. Recipients of the link with a subscription will also be able to download and print the PDF.

As soon as your article is published, you will receive an automated email with your shareable link.

You can now use a single sign-on for all your accounts, view the status of all your manuscript submissions and reviews, access usage statistics for your published articles and download a record of your refereeing activity for the Nature journals.

An online order form for reprints of your paper is available at https://www.nature.com/reprints/author-reprints.html. Please let your coauthors and your institutions' public affairs office know that they are also welcome to order reprints by this method.

If you have not already done so, we strongly recommend that you upload the step-by-step protocols used in this manuscript to protocols.io. protocols.io is an open online resource that allows researchers to share their detailed experimental know-how. All uploaded protocols are made freely available and are assigned DOIs for ease of citation. Protocols can be linked to any publications in which they are used and will be linked to from your article. You can also establish a dedicated workspace to collect all your lab Protocols. By uploading your Protocols to protocols.io, you are enabling researchers to more readily reproduce or adapt the methodology you use, as well as increasing the visibility of your protocols and papers. Upload your Protocols at https://protocols.io. Further information can be found at https://www.protocols.io/help/publish-articles.

Sincerely,

Wei Li, PhD
Senior Editor
Nature Genetics
www.nature.com/ng

Click here if you would like to recommend Nature Genetics to your librarian
http://www.nature.com/subscriptions/recommend.html#forms

** Visit the Springer Nature Editorial and Publishing website at www.springernature.com/editorial-and-publishing-jobs for more information about our career opportunities. If you have any questions please click here.**

Open Access This Peer Review File is licensed under a Creative Commons Attribution 4.0 International License, which permits use, sharing, adaptation, distribution and reproduction in any medium or format, as long as you give appropriate credit to the original author(s) and the source, provide a link to the Creative Commons license, and indicate if changes were made.
In cases where reviewers are anonymous, credit should be given to 'Anonymous Referee' and the source.
The images or other third party material in this Peer Review File are included in the article's Creative Commons license, unless indicated otherwise in a credit line to the material. If material is not included in the article's Creative Commons license and your intended use is not permitted by statutory regulation or exceeds the permitted use, you will need to obtain permission directly from the copyright holder.
To view a copy of this license, visit https://creativecommons.org/licenses/by/4.0/

Fast and flexible joint fine-mapping of multiple traits via the Sum of Single Effects model: Response to reviews and a summary of the revisions

Yuxin Zou[1,2], Peter Carbonetto[3], Dongyue Xie[1], Gao Wang[4] & Matthew Stephens[1,3]*

[1]Department of Statistics, University of Chicago, Chicago, IL, USA.

[2]Regeneron Genetics Center, Regeneron Pharmaceuticals, Inc., Tarrytown, NY, USA.

[3]Department of Human Genetics, University of Chicago, Chicago, IL, USA.

[4]Gertrude. H. Sergievsky Center, Department of Neurology, Columbia University, New York, NY, USA.

*e-mail: wang.gao@columbia.edu; mstephens@uchicago.edu

We thank the reviewers for the detailed comments on our manuscript. We have made the following improvements to the manuscript to address the reviewers' comments:

- We added more 2-trait simulations to compare the methods (SuSiE, mvSuSiE, CAFEH, PAINTOR, flashfm) in a greater variety of conditions (independent traits with independent effects, independent traits with correlated effects, correlated traits with independent effects). These simulations show that both flashfm and mvSuSiE improve power over SuSiE, but mvSuSiE consistently achieves greater gains in power than flashfm. These simulations also show that mvSuSiE is consistently better than CAFEH when the two traits are correlated.

- We performed functional enrichment analyses of the SuSiE and mvSuSiE results using GREGOR and gchromVAR.

- We added histograms to Fig. 2 showing the distributions of CS sizes for the different methods (SuSiE, mvSuSiE, CAFEH) in each of the simulation scenarios.

- We expanded the Discussion to address the points raised by the reviewers.

More details about these improvements are in the responses to the reviewers' comments below. We also made several other smaller changes throughout. To draw attention to these changes, we have highlighted **in blue** the parts of the main text that have changed substantially in the revised manuscript.

Reviewer 1

Remarks to the Author: In this manuscript, Zou et al developed and evaluated a novel multi-trait fine-mapping method, mvSuSiE. This method was an extension of a published and well-received single-trait fine-mapping method, SuSiE, which was also developed by the authors' team. I find mvSuSiE solid, thoroughly evaluated, and timely given the increasing global biobank

efforts. I especially like the thoughts and novelty in summarizing the inference across traits. The manuscript was well written. I have a few comments for the authors to consider.

Our response: We thank the reviewer for taking the time to review our work and provide feedback. We respond to the reviewer's individual comments in the paragraphs below.

Comment 1 (major): Throughout the manuscript, the authors used an empirical threshold of *lfsr* < 0.01. How was it determined? The authors claimed to find genetic associations with many blood traits in the EXT1–SAMD12 locus that the previous GWAS failed to find (Fig. 4A). But the finding was really based on this 0.01 threshold that was not justified (while GWAS used the 5e-8 “Bonferroni” threshold). In a sense this is a bit unfair. Could the authors set this threshold using a principled approach?

Our response: The reviewer is correct that the GWAS *p*-value and mvSuSiE *lfsr* thresholds are not comparable. Indeed, although we do mention the results from the single-trait GWAS, the important comparison here is between SuSiE (which identifies 1 CS for only 1 trait) and mvSuSiE (which identifies 1 CS that is significant in at least one trait). We made a small change to the text to make it more clear that we are only comparing the SuSiE and mvSuSiE results. The reason for mentioning the GWAS *p*-values in this paragraph was mainly to provide some intuition for univariate vs. multivariate analyses; for example, the fact that all traits had marginal *p*-values less than 0.003 provides *suggestive evidence* for association with all traits, but of course when each trait is considered in isolation without consideration of the other traits — which is what one would typically do in a genome-wide association analysis — a *p*-value of 0.003 is far from the standard “genome-wide” threshold (5e-8).

Regarding the choice of *lfsr* threshold, any choice of threshold is, inevitably, somewhat arbitrary; in frequentist significance testing based on *p*-values, the “standard” threshold choices of p = 0.05 and p = 0.01 are both arbitrary, and the GWAS threshold of 5e-8 is similarly arbitrary (at best, it is based on p = 0.05 plus some adjustment, and the choice of 0.05 is arbitrary). The *lfsr* (as well as *lfdr* and FDR) have an advantage over *p*-values in that they are estimates of an error rate, and a threshold of 0.01 should ensure this error rate is small (<0.01).

Comment 2 (major). The authors mentioned a limitation that mvSuSiE requires traits to be measured on all samples. The authors also mentioned “a general approach to deal with missing data” could potentially alleviate this limitation. I wonder if the authors could comment a bit more in this space. This limitation will preclude a lot of applications for mvSuSiE. For example, many disease genetic studies share the same control but have individuals diagnosed with different but related disorders that can be mutually exclusive (e.g., schizophrenia and bipolar). Do the authors have any recommendations for such studies? If mvSuSiE shouldn’t be used for these studies, perhaps the authors should make it clear to prevent misuse.

Our response: We have added a more detailed discussion on missing data, and the kinds of settings the reviewer mentions, to the revised manuscript (see “Discussion”).

Comment 3 (major): The correlation structure (U) was learned genome-wide from top SNPs. However, effect size correlation can sometimes be local (e.g., as discussed in several local genetic correlation papers). I wonder if the authors would comment on the implication.

Our response: We believe it is important to emphasize that the mvSuSiE prior is not characterized by a single correlation structure (U), but is a *mixture of multivariate normals*, each with its own distinct correlation structure (U_1, …, U_K). Thus, although the prior is learned by combining information across the whole genome, the use of a mixture prior allows that there may be different patterns of association at different loci, or even at different SNPs within a locus. So in this sense mvSuSiE can capture different patterns of correlation at different loci. We tried to explain this feature in the "Methods Overview":

> mvSuSiE uses a flexible prior distribution—a mixture of multivariate normal distributions—to model effect sharing patterns across traits. Further, the parameters of this prior are estimated from the data, allowing mvSuSiE to adapt to each data set. This flexible approach allows that different causal SNPs may show different patterns of association; for example, in analyses of blood cell traits shown later, mvSuSiE learns that some SNPs may affect primarily red blood cell (erythrocyte) traits, some may affect primarily white blood cell (leukocyte) traits, and some may affect both, or a subset of one or the other.

Comment 4 (major): I wonder if the authors could comment on applying mvSuSiE to case-control studies. And what if the summary statistics are generated from a linear-mixed model such as REGENIE rather than the linear model as used in the UKBB example (as LMM has gained much popularity with biobank studies)?

Our response: We have added a paragraph on this to the Discussion ("mvSuSiE assumes a standard (Gaussian) multivariate linear model…").

Comment 5 (minor): Lines 436–441: it would be helpful to give a rationale and intuition for *clfsr*.

Our response: First, to clarify, we note that the *clfsr* is not directly used in the mvSuSiE results, but is only used to calculate other quantities; in particular, it is used in calculating the "minimum *lfsr*" (*min-lfsr*) for assessing which traits are affected by the (putatively causal) SNP. In the Online Methods, under "Basic posterior quantities," we wrote the following, citing both Urbut et al (2019) and Stephens (2016):

> Intuitively, the *clfsr* ("conditional lfsr") measures how confident we can be in the sign of the effect of SNP *j* in trait *r* and single effect *l* given that SNP *j* has a nonzero effect in single effect *l*. A small *lfsr* indicates high confidence in the sign of an effect. The *lfsr* is robust to modeling assumptions which is helpful for reducing sensitivity to the choice of prior.

More detailed motivation for the *lfsr* is given in Stephens (2016), and how the *lfsr* was used in Urbut et al (2019) has guided our analysis choices here. If the reviewer feels that additional

rationale or intuition is needed, we would be happy to rectify this so long as the reviewer can explain what more specifically is lacking in our description.

Comment 6 (minor): Lines 146–148: I think the authors meant the “random effects prior” when saying “forced mvSuSiE to make the same independence assumptions as CAFEH”. However, Supplementary Figures 1–2 don’t fully support what the authors said in the text: “random effects prior” still appear to outperform CAFEH in Scenario b, e.g., in the coverage and median size.

Our response: This remark touches on two modeling aspects: an assumption that the effects are independent (“random effects”); and an assumption that the trait measurement errors are independent (“independent traits”). We considered these two aspects independently in our experiments. We have clarified this in the revised text:

> Comparing CSs (Fig. 2C), CAFEH improved the purity of the CSs and the proportion of 1-SNP CSs, but these improvements were tempered by CAFEH's reduced power and coverage, particularly in Scenario b. A partial explanation for these results is that Scenario b contradicts CAFEH's assumptions of independent traits and independent causal effects. In support of this explanation, when we forced mvSuSiE to make the same independence assumptions as CAFEH, mvSuSiE's performance was reduced and the PIPs were also poorly calibrated (see the “random effects prior” and “independent traits” results in Supplementary Figures 1, 2, 9).

Comment 7 (minor): Didn’t “1-SNP CS” and “median of purity” panels in Fig. 2C show that mvSuSiE is doing worse than CAFEH?

Our response: The reviewer is correct that one would prefer CSs with higher purity. Indeed, the median purity of mvSuSiE is 0.998 compared to 1 for CAFEH. (Note that the Y axis was shortened in this plot to show the difference, so the difference is actually quite small.) Similarly, CAFEH identifies more 1-SNP CSs than mvSuSiE. However, these improvements are outweighed by CAFEH's considerable reduced power and coverage. The drop in coverage is particularly glaring in Scenario b. We added a sentence to the results to clarify this: “Comparing CSs (Fig. 2C), CAFEH improved the purity of the CSs and the proportion of 1-SNP CSs, but these improvements were tempered by CAFEH's reduced power and coverage, particularly in Scenario b.”

Comment 8 (minor): Fig. 3 legend needs more details, e.g., how was the proportion of CS in panel a calculated?

Our response: For N traits, the height of the bar in Fig. 3A is simply the number of CSs that are significant for N traits divided by the total number of CSs. The meaning of “proportion” is unambiguous in this context, and so we have not given further explanation in the figure caption. But if there are any aspects to this figure in particular that you feel require further explanation, please tell us specifically what is unclear and we will do our best to clarify.

Comment 9 (minor): It can be helpful to add the grouping of blood cells in Fig. 4, e.g., what are the cell types that are “mature red blood cells”. I understand the authors might have included this info, but it’d be good to show it with the figure to make it easier for the readers.

Our response: Thank you for this suggestion. We have added this information to Fig. 4.

Comment 10 (minor): Perhaps the authors can do a better job connecting Fig. 1 to the methods section. For example, the authors could make it clear that Panel C is related to the approach for estimating V (eq. 24) and e for estimating U (cite the discussions in the prior).

Our response: Thank you for this suggestion. We now connect the different parts of Fig. 1 to the relevant sections of the Online Methods.

Comment 11 (minor): Is I_R in the canonical prior the same as “random effects prior”? If so, perhaps the authors should remove the random effects prior (lines 485–488) and use only the remaining two categories of priors (canonical and data-driven) to avoid confusion.

Our response: The reviewer is correct that the identity mixture component in the canonical prior models is the “random effects” prior (the prior that assumes all effects are independent). However, the random effects prior and canonical prior are very different: the canonical prior is a *mixture* of simple sharing patterns, which can model the sharing of effects across conditions, whereas the random effect prior does not. We tested mvSuSiE with the random effects prior because other methods (e.g., CAFEH) also use this prior (see the “models effect sharing” column in Table 1), and therefore including these tests would help us understand better how the random effects prior affects performance. This was already described in the Online Methods (see “Specifying the prior”), so we did not change the text.

Comment 12 (minor): Supplementary Fig. 5: Why well-calibrated methods should have points on the diagonal line? I understand if it’s under null for a Q-Q plot but I thought this is something different.

Our response: The reviewer's confusion may be due to a mistake in the caption. The caption should read: “The plots show the average PIP from each bin (X axis) against the proportion of SNPs in that bin that are causal (Y axis).” We have updated the caption to correct the error (now Supplementary Fig. 9). We apologize for the confusion.

Comment 13 (minor): Supplementary Figures 6–9: could the authors label each panel for its underlying covariance matrix (equal effect, *etc*)?

Our response: We have added labels to Supplementary Figures 6–9 (now Supplementary Figures 10–13) as suggested.

Reviewer 2

Remarks to the Author: Zou et al present mvSuSiE, a multivariate extension of the ultra-scalable “Sum of Single Effects” fine-mapping model that seeks to jointly fine-map multiple traits/outcomes. While there exist methods for fine-mapping multiple traits jointly, they either fail to scale to large datasets, or do not leverage a shared architecture assumption, which may lose power when it exists. mvSuSiE addresses these limitations by incorporating the SuSiE model for fast inference, while using multivariate priors on effect-sizes across traits that allow for correlations to exist *a priori*. The authors validate their approach in simulations and analyze large-scale GWAS of blood traits in the UK Biobank. I found the manuscript well written and timely. With that said, I have some comments.

Our response: Thank you for taking the time to review our work and comment on it. We have responded to your individual comments in the paragraphs below.

Major Comment 1: The authors performed extensive simulations that were informed from real data scenarios. While this helps understand behavior in typical use cases (which is important), I found it hard to parse with respect to specific modeling aspects.

It would be helpful to see how mvSuSiE performs (or outperforms) as residual correlations increase, or similarly as correlations in effect sizes increase. This would provide helpful information to see the extent of performance along a gradient of scales.

Similarly, how much of the improvement of mvSuSiE comes from refinement over mvIBSS results?

Our response: To address the first part of this comment, we performed a new set of simulations with 2 traits in which we varied the correlation among the measurement errors. (We also ran more simulations with 2 traits in which varied the correlation among the effects; see our response to Major Comment 1 from Reviewer 3.) In this simulation, we compared the fine-mapping performance of mvSuSiE with SuSiE, CAFEH and flashfm. The results of these comparisons are summarized in Supplementary Figures 5 and 6. As expected, detection of cross-trait causal SNPs (power) improved as the measurement errors were more correlated, but mvSuSiE showed substantial gains in power whereas CAFEH only show a slight improvement. This was expected because CAFEH assumes that the measurement errors are independent, whereas mvSuSiE does not make this assumption.

We apologize, but we don't understand the last part of this comment. By “mvIBSS”, we are guessing that the reviewer is referring to the mvSuSiE IBSS algorithm, which is described in the Supplementary Note. This is the algorithm used fit an mvSuSiE model, so it doesn't make sense to compare mvSuSiE to the IBSS algorithm—the IBSS algorithm *is* the algorithm used to fit the mvSuSiE model.

Major Comment 2: I was excited to see the real-data results and their interpretations but found myself underwhelmed with the results as reported. The authors describe the improvement in credible sets identified and single-SNP credible sets, but it is less clear if the method is

discovering novel biological signal, or if it is a consequence of false positives on average. The examples listed certainly help, but a more broad analysis would place the general results in a clearer light. One possible way to dissect this (albeit with its own flaws) would be to perform functional enrichment using trait/cell matched annotations. Are cell/trait matched functional annotations more enriched for PIPs/CS signals from mvSuSiE than those inferred from SuSiE?

Our response: Regarding whether the additional fine-mapped SNPs identified by mvSuSiE are due to false positives, we view the simulation results (where ground truth is known) as the best, albeit imperfect, way to support that mvSuSiE is not expected to produce large numbers of additional false positives. Nonetheless, following the reviewer's suggestion, we also performed two sets of enrichment analyses using the SuSiE and mvSuSiE fine-mapping results for the blood cell traits. Overall, the mvSuSiE-fine-mapped SNPs showed slightly stronger enrichments in functionally relevant features (e.g., greater enrichment for eQTLs), supporting that they are not false positives. The hematopoietic cell-type enrichments, which followed an analysis similar to Vuckovic et al (2020), showed some of the expected enrichments—e.g., enrichment of SNPs affecting platelet-related traits for open chromatin in platelet-producing megakaryocytes—but it was generally unclear whether these enrichment results indicated an improvement of mvSuSiE over SuSiE.

Major Comment 3: Similarly, the authors focus on comparisons between CAFEH and SuSiE in simulations, but only compare with SuSiE in real data analysis. While comparison against SuSiE is helpful to see gains due to multivariate assumptions, it would also help to assess gains due improved modeling assumptions in mvSuSiE vs CAFEH (e.g., non-independence of traits).

Our response: Our main aim in comparing SuSiE vs. mvSuSiE here was to illustrate the impact of a multivariate fine-mapping analysis—in contrast to a more typical univariate fine-mapping analysis—on real data. From the simulations, we found that CAFEH had elevated false positive rates, particularly when the traits were correlated (Fig. 2). Since the blood cell traits are correlated, and some quite strongly (see Supplementary Table 5, which gives the estimate of the residual correlation matrix), our concern is that CAFEH would produce misleading results.

Minor Comment 1: No color legend is supplied for Fig 3E.

Our response: Thank you, we have added the missing legend to Fig. 3E.

Reviewer 3

Remarks to the Author: The authors extend SUSIE for single-trait fine-mapping to multiple traits, mvSuSiE. It can be used with individual-level or summary data, but requires all traits to be measured on each individual in the cohort (*i.e.*, no missing trait measurements). Simulations show that 20 traits can be jointly fine-mapped, and they use mvSuSiE to jointly finemap signals from 16 blood cell traits in UK Biobank. Some detailed comments follow.

Our response: Thank you for reviewing our work and providing detailed feedback. We have responded to your individual comments in the paragraphs below.

Major Comment 1 (lines 116–117): The simulation comparison between mvSuSiE, PAINTOR, and flashfm is incomplete. A more realistic scenario with two correlated traits (rather than independent) that share at least one causal variant, though not all shared, is needed. Also, credible set sizes should be compared for all methods.

Our response: To address this comment, we performed a new set of simulations with 2 traits in which we varied how the effects were simulated, from effects being independent to effects being strongly correlated, as well as simulations that had a mixture of independent and correlated effects. (We also performed additional simulations with 2 traits in which we varied the correlation among the measurement errors; please see our response to Major Comment 1 from Reviewer 2.) In these simulations, we compared the fine-mapping performance of mvSuSiE with SuSiE, CAFEH and flashfm. The results of these comparisons are summarized in Supplementary Figures 7 and 8. In this particular setting, the mvSuSiE and CAFEH models are very similar, so it is not surprising that mvSuSiE and CAFEH have similar performance in identifying cross-trait causal SNPs. For detecting trait-specific causal SNPs, flashfm continues to perform poorly in these simulations, consistent with our previous results (Supplementary Fig. 3).

Major Comment 2 (lines 139–140): Proportion of 1-SNP credible sets is given for mvSuSiE and CAFEH comparisons, but it is also important to consider the size of these sets when there is more than one SNP in the set. Please include a comparison of credible set sizes for these methods.

Our response: Thank you for the suggestion. Fig. 2 now has histograms showing more detail about the CS sizes.

Major Comment 3: A discussion is needed on the trade-off between joint analysis of many traits in a reduced sample size compared to a joint analysis of fewer traits in a larger sample or even a single trait in a larger sample. Their UK Biobank analysis had n ~ 250k, where all 16 traits were measured in the individuals, but the previous analysis by Vuckovic et al. had n ~ 408k. It would be helpful to include an application of SUSIE to the complete UK Biobank data for some of the highlighted examples.

Our response: Thank you for these suggestions; we have added some text to the Discussion about this tradeoff and more generally about dealing with missing data.

To clarify the point about sample size, we could not find details from Vuckovic et al (2020) about the exact filtering steps taken, nor exactly how they ended up with 408,112 samples. We removed related individuals (based on kinship calculations) and samples that were not labeled as "White British". These two steps removed, respectively, 126,236 and 92,887 samples, so the large difference in sample size between us and Vuckovic et al (2020) is probably due to different data preparation steps, and not due to missing blood cell trait measurements. More details

about the data preparation are provided in the Online Methods (“UK Biobank data”), and the exact steps we took are documented in this R script, https://tinyurl.com/ydtt8855 (which is included in one of the Zenodo repositories listed in “URLs”).

Minor Comment 1 (line 25): Cite which methods are limited to fine-mapping only two traits.

Our response: We revised this sentence so that we no longer mention methods that are limited to only 2 traits.

Minor Comment 2 (line 25): Cite which methods are limited by 1–2 causal variants.

Our response: We revised this sentence so that we no longer mention methods that are only limited to 1–2 causal variants since few methods had this limitation (PAINTOR was one of the methods with this limitation).

Minor Comment 3 (Table 1): Add a column for upper limit on the number of traits for each method.

Our response: We initially had a column for this in Table 1, but later we removed it. Most methods can, in principle, handle many traits, but in practice methods are limited by computational considerations. As far as we are aware, only flashfm has an explicit constraint on the number of traits.

Minor Comment 4 (lines 59–67): The other multi-trait methods (PAINTOR, flashfm) that are in the simulations should be discussed here.

Our response: We compared mvSuSiE to CAFEH because they have many common features. PAINTOR and flashfm are quite different to mvSuSiE and thus less helpful for explaining the benefits of mvSuSiE. PAINTOR and flashfm also have important limitations in which multi-trait data sets they can be applied to (see Table 1).

Minor Comment 5 (line 153): Please provide the details of the set of simulations that compare mvSuSiE with PAINTOR and flashfm. How many SNPs in the region, how many causal SNPs, how many shared causal SNPs?

Our response: Since space is limited in the main text, these details were provided in the Online Methods (see “Simulations using UK Biobank genotypes”). All the simulations used the same set of 975 fine-mapping regions. We also revised the description in the main text slightly to clarify that the 2-trait simulations are similar to simulation scenarios “a” and “b”.

To answer your question about how many causal SNPs were shared across traits, all causal SNPs were shared by all traits, but the effects of the causal SNPs on the traits were simulated in different ways depending on the simulation scenario. In the 2-trait simulations, the effects on the two traits were simulated independently to align with PAINTOR's modeling assumptions. (In

the main text, we wrote, “Even when the traits were simulated independently in accordance with PAINTOR's modeling assumptions, PAINTOR had much lower power to detect causal SNPs than both SuSiE and mvSuSiE.”

Minor Comment 6 (Supplementary Fig. 4): It is difficult to see the details for all methods, so please list the median (and quartiles) running times for all three methods.

Our response: These numbers are now provided in Supplementary Table 1.

Minor Comment 7 (lines 707–709): This statement is confusing and could cause errors when running flashfm. Users will get incorrect results if they input a zero vector for the effect allele frequencies (EAF) to any of the flashfm wrapper functions, or to the flashfm function if the trait means are non-zero.

Our response: We agree that this statement could be confusing. Since we aren't fully confident about this statement, we have removed it from the paper.

Fast and flexible joint fine-mapping of multiple traits via the Sum of Single Effects model: response to reviews and a summary of the revisions

Yuxin Zou, Peter Carbonetto, Dongyue Xie, Gao Wang and Matthew Stephens

We thank the reviewers for their detailed comments on our manuscript. We have made the following additional changes to the manuscript to address the reviewers' comments:

• For the mvSuSiE results in the simulations, we now provide results (Fig. 2, Supplementary Table 1, Supplementary Figures 1, 7, 10) at two different *lfsr* thresholds to illustrate how different *lfsr* thresholds impact different quality measures (power, FDR, coverage, *etc*).

• In all simulations, we now provide histograms giving a more detailed comparison of the credible set (CS) sizes (Fig. 2, Supplementary Figures 6, 7, 9, 10).

• We added some text clarifying the presentation of the simulation results in Fig. 2.

• We updated the Discussion slightly to clarify potential extensions of mvSuSiE, in particular for analyzing data from multiple studies ("meta-analysis fine-mapping").

More details about these changes are in the responses to the reviewers' comments below. We have also made several other smaller changes throughout. To draw attention to these changes, we have highlighted in **blue** the parts of the main text that have changed substantially in the newly revised manuscript.

Reviewer 1

Remarks to the Author: The authors have addressed most of my concerns. mvSuSiE is a novel, solid, and very timely fine-mapping method. My remaining questions mainly focus on improving the interpretation of the results (follow up of the previous comment 1) and the method's utility (follow up of the previous comment 2).

Our response: Thank you again for your constructive review. We have responded to your individual comments in the paragraphs below.

Interpretation of results (follow up of Comment 1): I agree with the authors that a choice of threshold is arbitrary, and mvSuSiE is a Bayesian method that estimates the probability, which differs from the frequentist approach. However, I found the interpretation of *lfsr* unclear, which prevented mvSuSiE's utility as a Bayesian method. For example, the authors said, "*lfsr*… are estimates of an error rate" in the response; what is the error *lfsr* measures? The manuscript defines *lfsr* as "how confident we can be in the sign of the effect." How does *lfsr* quantify "confidence"? For example, can *lfsr* be interpreted as the probability that the CS is not causal to

trait *r*? The author brought up FDR and P-value in the response, I think giving *lfsr* a clear definition as FDR and P-value would be very helpful.

Regarding the threshold, indeed, any threshold is arbitrary. However, future studies using mvSuSiE will likely use the 0.01 threshold the author used in the manuscript. I'd like to see if this threshold can be justified as reasonable for future studies.

Our response: To directly answer the question, "what is the error *lfsr* measures?", an error is defined as getting the sign of an effect wrong. Note that if an effect is truly zero then finding an effect of either sign (positive or negative) is an error. To draw an analogy to the *local false discovery rate* (*lfdr*), getting a low *lfdr* requires one to correctly identify the non-zero effects; getting a low *lfsr* requires one to correctly identify the non-zero effects *and to get their signs right*. Thus, *lfsr* is more conservative than *lfdr* because its notion of error is more expansive (anything counted as a false discovery is an error, but true discoveries are also errors if their signs are wrong).

To directly answer the question, "How does *lfsr* quantify confidence?", it uses the posterior probability. So the *lfsr* is the posterior probability that the sign of the true effect is different from the estimate.

Again, the *lfsr* is more stringent than the *lfdr* because it requires true discoveries to be not only nonzero but also correctly signed. In fact, *lfsr* ≥ *lfdr*, and in this sense, the *lfsr* is a more conservative measure of significance than the *lfdr*. However, the real advantage of *lfsr* over *lfdr* is that it is more robust to modeling assumptions, which is helpful in an mvSuSiE fine-mapping analysis where we would like to be robust to inaccuracies in the estimated sharing patterns. Also, the *lfsr* does not require that the null effects be exactly zero, which is arguably important in genetic applications where it is often expected that no genetic effects are exactly zero (say, due to population structure or highly polygenic traits). For further information on *lfsr* and its connection with FDR and p-values, please see Stephens (2017) and Stephens (2023).

The precise definitions of the *lfsr* measures we used are all given in the Online Methods. We have added some clarifying sentences near these definitions to try to make the intuition behind these definitions clearer. We have also added a sentence to the main text clarifying that a small *lfsr* indicates a small posterior probability that the true effect is zero or that its estimated sign is incorrect.

To address the suitability of *lfsr* < 0.01 threshold, we have added results for this threshold to our simulation studies (Fig. 2). Compared with the 0.05 threshold, the 0.01 threshold naturally has slightly lower FDR (B) and better coverage (D) but at the cost of slightly lower power. Results at both of these thresholds are also included in the supplementary figures. Also see the new Supplementary Table 1. In our view, both thresholds give acceptable error rates (Fig. 2B) and coverage (Fig. 2D) for practical application (e.g., coverage exceeds 0.95 for both thresholds), but in the analysis of the UK Biobank blood cell traits we used the more stringent *lfsr* threshold of 0.01 to encourage the use of more stringent thresholds in future studies.

Utility (follow up of Comment 2): The authors mentioned in Discussions that “The multivariate linear model (1) assumes that the traits are measured in the same samples”. How bad if this assumption is violated? Summary statistics from consortia (e.g., Psychiatric Genomics Consortium) can have highly related traits generated on datasets from different samples, often with overlaps. Clarifying this can make sure mvSuSiE is used appropriately in such cases.

Our response: While this is an interesting question, it is not directly addressed in our paper, and so we cannot say much about it. We did touch on this point in the Discussion (see the last paragraph), and we have revised our discussion to provide more detail and clarification. As we say in the paper, yes indeed, there is the potential to extend mvSuSiE to handle such situations. However, the existing mvSuSiE model has assumptions that would make it inappropriate for analyzing data from multiple studies ("meta-analysis fine-mapping"). The question of when (or whether) it is appropriate to apply *existing single-trait fine-mapping methods* to summary statistics from meta-analyses is studied in a recent paper by Kanai et al in *Cell Genomics* (we cite this paper in our manuscript). One key takeaway from this paper is that existing fine-mapping methods do not account for differences among the studies (e.g., differences in sample size), and not accounting for differences can lead to miscalibrated fine-mapping results. Therefore, although there is clearly interest in performing fine-mapping using summary statistics from consortia (as you highlight), we would urge caution in proceeding with such analyses without appropriate statistical tools.

Reviewer 2

Remarks to the Author: The authors have addressed my original comments. I have no new comments at this time.

Our response: Thank you!

Reviewer 3

Remarks to the Author: The authors have not addressed all comments, and vague and misleading answers have been provided. All simulation comparisons need more explanations/details.

Our response: We thank the reviewer for reading our revised manuscript and commenting on the changes. We certainly did not intend to provide misleading answers, and we have tried to improve our responses in this response. We respond to the reviewer's individual comments in the paragraphs below.

Comment 1: The simulation comparison of mvSuSiE with PAINTOR and flashfm is still incomplete. Although the authors now include comparisons where the traits are correlated, they have not used a realistic setting where there are multiple causal variants for each trait and not

all causal variants are shared between the traits (as suggested). The two-trait simulation is still for only causal variants shared by both traits.

Our response: We did in fact include such simulations, although our description of these simulations was perhaps unclear in our previous response. The results from these simulations are shown in the bottom row, labeled "mixture", in Supplementary Figures 8–10. In these simulations, we simulated the effects on the 2 traits from a mixture of 7 "canonical" covariance matrices. These covariance matrices include (i) independent effects, (ii) heterogeneous effects and (iii) trait-specific effects. Both (i) and (ii) simulate SNPs that are causal for both traits, and (iii) simulate SNPs that are causal for only one of the two traits. The full details were given in the Online Methods (see in particular "Simulation scenarios" and "Simulation procedure").

Comment 2: Are the cross-trait PP not used at all for trait-specific mvSuSiE results? I was under the impression that trait-specific variants should have high cross-trait PP (so causal for at least one trait) with small *lfsr* for the particular trait, but this does not seem to be the case in the trait-specific simulations. The authors must explain how to identify causal SNPs and which traits are affected by them and then evaluate this approach in their trait-wise simulation comparisons with CAFEH and flashfm.

Our response: We assume that here, and in subsequent comments, "cross-trait PP" is a typo, and the reviewer meant "cross-trait PIP".

The reviewer's intuition is correct: trait-specific variants should have both high cross-trait PIP and small **conditional** *lfsr* (*clfsr*) for the particular trait. The *minimum lfsr*, which we use in the trait-specific simulations (results in Fig. 2B), essentially combines information in the cross-trait PIP and the *clfsr* into a single number.

To make this statement more precise, consider the case of a single causal effect (L = 1). In this case, *min-lfsr* = *lfsr* $= 1 - \alpha(1 -$ *clfsr*) [see eq. 22 in the Online Methods]. In the case of L = 1, α is exactly equal to the cross-trait PIP (eq. 19 in the Online Methods). From this equation, one can see that indeed *lfsr* is small only if the cross-trait PIP (α) is large and the *clfsr* is small. For L > 1, each l = 1, …, L captures a different signal, and so we compute *lfsr* separately for each l and take the minimum of *lfsr* across l as an overall measure of significance (eq. 22 in the Online Methods).

Regarding the comment, "the authors must explain how to identify causal SNPs and which traits are affected by them," in the "Methods overview" we tried to be clear how a typical mvSuSiE analysis should proceed: see the paragraphs starting with "First question" and "Second question". To further improve this explanation we have added additional text ("In practice, we recommend focusing on CS-based inferences…" under "Evaluation in simulations using UK Biobank genotypes") to emphasize that we recommend focussing on the CSs rather than individual SNPs because strong LD can cause all SNPs in a CS to have small PIPs (exactly the problem that the CSs are designed to address). We note that this is exactly how the mvSuSiE fine-mapping analyses proceeded for the UK Biobank blood cell traits. Fig. 2C and D directly

assess the operating characteristics of this recommended mvSuSiE pipeline. However, although our recommended pipeline focuses on CS-based inferences, other methods do not provide measures of significance of each CS in each trait, so we could not compare other methods in Fig. 2D. This is why we included the SNP-based results in Fig. 2B. Again, our new additional text (“In practice, we recommend focusing on CS-based inferences…”) aims to clarify this.

Comment 3: The trait-specific power-FDR plots are misleading because they vary thresholds for two very different measures, PIP/MPP (for CAFEH, SuSiE, flashfm) and *lfsr* (for mvSuSiE). The only disclosed thresholds are at baseline (open circles) with a more lenient *lfsr* threshold of 0.05 compared to stringent PIP/MPP thresholds of 0.95; in the data application, the authors used *lfsr* threshold 0.01 and PIP threshold 0.95, and they should display both in simulation comparisons. Also, as detailed above, it needs to be clarified whether thresholds for cross-trait PP and *lfsr* are used together in mvSuSiE for trait-wise results—this does not seem to be the case in the simulations.

Our response: Regarding the results shown in Fig. 2B (as well as the other power vs. FDR plots on detection of trait-wise causal SNPs), the reviewer is correct that the significance measures and thresholds used are not directly comparable. But this does not mean the plots are misleading; they allow one to compare, for example, the power (shown on the Y axis) at a given FDR (X axis). Such plots are commonly used to compare measures that are very different from one another (e.g., Bayes Factors and p-values). As requested, we have updated Fig. 2 to show power and FDR at *lfsr* thresholds of 0.01 and 0.05 (as well as PIP thresholds of 0.99 and 0.95 for other methods that do not provide *lfsr*). Also see the new Supplementary Table 1 giving the exact numbers corresponding to what is shown in Fig. 2B (we provide this table since the plots are quite small and therefore small differences are hard to distinguish).

As we explain in the response to the previous comment above, the *min-lfsr* used in mvSuSiE essentially combines information used in the cross-trait PIP and *clfsr* into a single number so there is just one threshold being used, not two thresholds being used together. (This applies to both the simulations and the real-data analysis.)

Comment 4: Figure legends lack details and they should contain enough information to understand the comparisons made in each figure. It is not clear how each plot is produced, power definitions, specific thresholds for each method, *etc.* The narrative of the paper and key takeaways are based on such pictorial representations.

Our response: In the revised manuscript, we have added more detail to Fig. 2 and the supplementary figures (Supplementary Figures 1, 7, 10) about the thresholds used. We have already provided definitions of power, FDR, coverage and purity in the caption for Fig. 2. Since the figure legends are already quite long and detailed, it is not feasible to give much more detail beyond what we have already given in the captions. We would also like to point out that the code and data used to generate the results in the paper are provided in Zenodo repositories (see "URLs"). If there are specific definitions or other important technical details that you believe

are unclear or are missing from the main text, the Online Methods, or the supplementary text, please let us know and we will be happy to correct the issue.

Comment 5: There are no convincing arguments of why *average lfsr* of 0.05 is used in some simulation comparisons instead of *lfsr* for each causal variant for a particular trait—the 0.05 threshold is also questionable. I see in the UK Biobank application that *lfsr* < 0.01 was used to determine the significant CSs.

Our response: Regarding *lfsr* vs. *average lfsr*, we note that *average lfsr* provides a way of assessing significance of a CS, whereas lfsr (and *min-lfsr*) assess significance of individual SNPs. As noted in responses above, we recommend focussing on the CSs rather than individual SNPs, and we believe the revised manuscript makes this clearer. This is why *average lfsr* is used in some simulation comparisons.

Regarding choice of threshold, we agree that the simulations should include the same threshold as in the real data analysis. The revised Fig. 2, Supplementary Table 1 and Supplementary Figures 1, 7 and 10 now include mvSuSiE results at *average lfsr* thresholds of both 0.01 and 0.05 to illustrate how different thresholds impact the quality of the results (power, FDR, *etc*).

Comment 6: Supplementary Figures 5, 6, 7, 8: median CS size is just a point estimate and not very informative without any bounds or indication of the distribution. Such bespoke choices require reasonable pieces of evidence to become good practice.

Our response: As requested, we have added histograms to these figures (now Supplementary Figures 6, 7, 9 and 10 comparing in greater detail the CS sizes.

References

M. Stephens (2017). "False discovery rates: a new deal." *Biostatistics* **18**, 275–294.

M. Stephens (2023). "The Bayesian lens and Bayesian blinkers." *Philosophical Transactions of the Royal Society A* **381**, 20220144.

MATTHEW STEPHENS, PH.D.
Professor
Departments of Statistics and Human Genetics
University of Chicago
email: mstephens@uchicago.edu
Tel.: (773) 834-5248

Dear Referees,

We have been invited by the editors of *Nature Genetics* to once again resubmit our manuscript, “Fast and flexible joint fine-mapping of multiple traits via the Sum of Single Effects model”, (NG-TR62293R4), and respond to the reviewer comments. Please see below for the original reviewer comments and our responses, together with brief descriptions of the changes made to the manuscript to address these comments and pointers to the relevant passages of the text.

Sincerely,

Matthew Stephens

Reviewer 1's comments

I believe the authors have addressed Reviewer 3’s comments 1 and 2 effectively. The mvSuSiE method has a complex design, incorporating two measures: PIP and lfsr. While PIP is widely used in fine-mapping studies, lfsr is relatively novel and not commonly employed in most existing methods. As the authors have noted, lfsr is fundamentally different from PIP, which makes direct comparisons with other methods difficult or even infeasible.

Further complicating such comparisons is the fact that PIP values are not calibrated across fine-mapping methods, as noted in several publications (e.g., https://pubmed.ncbi.nlm.nih.gov/36643910/). In this context, I find the use of FDR to be appropriate and justified, as it has been used to align results across different fine-mapping approaches fairly.

Overall, I think the authors have done a good job responding to Reviewer 3’s comments 1 and 3. The thresholds are now aligned, and the manuscript includes clear notes cautioning readers about the limitations in comparing methods. One optional suggestion I have is to add a brief explanation in the Discussion section about the use of lfsr, particularly for readers who may not be methodological experts. As I mentioned previously, my concern is the potential misuse or misinterpretation of lfsr as a novel measure.

Regarding comment 2, it appears the authors did use the “stage 2 lfsr” in the UK Biobank analysis, as indicated in Line 242: “The number of CSs significant in each trait (average lfsr < 0.01).” This seems to contradict the statement that “these steps are not used in the UK Biobank analyses.” It would be helpful for the authors to clarify this point.

Finally, I noticed a minor error in this version: in eq. 23 of the supplement, the summation should be over SNPs (indexed by J), not the number of effects (L).

Our response to Reviewer 1's comments

Thank you very much for your comments. Regarding the use of the local false sign rate (*lfsr*), the use of the *lfsr* for analyzing multi-trait effects was introduced in our earlier work on multivariate adaptive shrinkage (Urbut et al, *Nat. Genet.*, 2019), so it is therefore natural to consider the use of the *lfsr* in the context of *multi-trait fine-mapping*. However, we also agree that the use of the *lfsr* for fine-mapping will most likely be unfamiliar to researchers who are already familiar with existing fine-mapping approaches. The use of the *lfsr* was extensively detailed and discussed in our previous work, and we are not sure that there is anything useful or interesting we have to add to the use of *lfsr* in the context of fine-mapping. This comment however did remind us of an important point that was explored in the current work: while we focussed on assessing the effects of the SNPs on traits *by sign*, there is also the potential to compare effects across traits in different ways, such as comparing the effects *by magnitude*, which is what we did in Urbut et al (2019), and might be especially useful for situations in which mvSuSiE is being applied to a large number of traits. We added a sentence about this in the Discussion.

Regarding the reviewer's comments about miscalibration of PIPs, we agree that this is an important—and perhaps underappreciated—issue, and the work by Kanai et al (2022) highlights this important issue. Broadly speaking, we believe that miscalibration arises from a discrepancy between the (implicit) modeling assumptions that are made by the fine-mapping methods and how they are used in practice with summary statistics. (In short, the computation of the summary statistics is often based on models that are not consistent with the fine-mapping methods' modeling assumptions.) In very recent work, we have also discovered that this miscalibration arises at small sample sizes, which is common in fine-mapping of eQTLs (Denault et al, *biorXiv*, doi:10.1101/2025.05.16.654543). Regardless, the reviewer is right to point out that miscalibration is an important issue generally, and was certainly an important consideration for this paper (in particular, the issue of CAFEH producing miscalibrated PIPs was mentioned in the main results). For this reason, the power-FDR curves were a helpful way to compare the performance of the PIPs in a way that was not compromised by miscalibration.

Regarding our statement "these steps are not used in the UK Biobank analyses," we believe that things became confusing due to the back-and-forth in the review process, and indeed it is possible that we misinterpreted the reviewer's comment. To explain our statement, it is important to note that there are two types of inferences that we consider in the paper: *SNP-based inferences* and *CS-based inferences*. Our interpretation of the reviewer phrase "stage 1 cross-trait PIP together with stage 2 *lfsr*" was that it referred to SNP-based inferences; we never used the phrases "stage 1" or "stage 2" in the manuscript, but the cross-trait PIP is inherently SNP-based, so this comment on "stage 2 *lfsr*" seemed to us to be about SNP-based inference. Our response to the reviewer was therefore intended to emphasize the fact that we did not

focus on SNP-based inferences in our UK Biobank analyses, only CS-based inferences. To ensure that this is completely clear, we added another note on this to the paper. The relevant sentence now reads: “In practice, we recommend focusing on CS-based inferences (Fig. 2C, D) rather than SNP-wise measures (Fig. 2A, B) because the CSs account for uncertainty in the causal SNP due to LD (and in our multi-trait fine-mapping of blood cell traits below, we focus on CS-based inferences).”

In any case, perhaps the most important part of Reviewer 3’s comment was “they do not address why they do not replicate these steps in their simulations. Simulations are used to understand how the methods perform when we know the truth, so it is customary to use the same approach in the data analysis as in the simulations.” As noted in our previous responses, we do indeed replicate the steps used in the real data analysis in our simulations: Specifically, *Figs. 2C and D assess the operating characteristics of the CS-based inferences, which are the inferences used in the UK Biobank data analysis.*

Finally, thank you for noticing the typo and reporting it to us. We have corrected this typo in the updated Online Methods.

Reviewer 2's comments

It has been some time since my initial review, but will add my remarks on the validity of points made by Reviewer 3 as well as the authors' response.

First, regarding the choice of thresholds and Power/FDR curves.

I find myself agreeing—to some extent—on the utility of Power/FDR curves in fine-mapping papers. While these curves have gained popularity in recent years, and are a staple in general statistical settings, I feel their inclusion has been somewhat detrimental to not only fine-mapping method developers but also consumers of these tools.

Namely, fine-mapping is a challenging variable selection problem, where our knowledge of the underlying biology has driven the development of statistical concepts to reflect this. For example, credible sets provide an interpretable (largely) means to capture putative causal variants after accounting for LD. Straightforward metrics in simulations based on sensitivity/calibration of PIPs and credible sets are helpful and maps back to how practitioners THINK AND USE these tools.

In contrast, Power/FDR curves may be overly conservative/unrealistic due to LD complications (i.e., single causal in perfect LD ≥ PIP of 0.5 would penalize these curves, but be completely reasonable in a CS metric), notions of variable thresholds, and when practitioners decide to fine-map in the first place.

Now with that said, I have no strong concerns about their use in simulations to present some notion of performance as they are still informative, but with their own limitations. Similarly, I am

sympathetic to the authors regarding minor typos or missed changes in supplement when a large number of supplemental materials exist, provided the authors make their best effort to correct/address this (which it seems they have).

The remaining concerns raised by Reviewer 3 seem to be a misunderstanding on their part, which is not due to a lack of information from the authors from what I can gather. Their approach was outlined in a previous submission which may have been missed by the reviewer.

Our response to Reviewer 2's comments

Thank you for your feedback on the latest iteration of this work. We agree very much with the reviewer's assessment of power-FDR curves: they are an informative but imperfect tool for assessing fine-mapping performance. We also agree that "*credible sets provide an interpretable (largely) means to capture putative causal variants after accounting for LD. Straightforward metrics in simulations based on sensitivity/calibration of PIPs and credible sets are helpful and maps back to how practitioners THINK AND USE these tools."* This is why we focussed on CS-based inferences in our real data analysis, and whby we highlighted several "straightforward metrics" on these CSs in our simulations (e.g., Fig. 2, Panels C and D).

Reviewer 3's comments

Transparency in results has been a consistent theme in my comments throughout the review process. From the early stages, I requested details on the thresholds used at various points in the plots to assess how the methods perform at typical analysis thresholds. Only in the fourth revision has the bare minimum level of transparency been met, with details now provided for two plausible and somewhat comparable thresholds. I understand the authors' frustration, but providing underlying data for plots is standard practice.

With this minimum level of transparency now in place, it becomes clear that the new approach offers no significant gains over existing multi-trait methods. The previous revision included two threshold points, but one was an unrealistically stringent PIP > 0.99 for current methods—something not used in practice and now removed from this revision.

There is a discrepancy between implied outperformance and tangible evidence-based outperformance of the proposed method over the existing ones. The narrative of the paper is that the proposed method outperforms the existing methods, and the method comparison visualizations give the impression of even larger improvements. For example, the plots exaggerate differences by focusing on thresholds not used in practice. Supplementary tables show FDR is generally <0.05 at lenient thresholds (PIP > 0.80, lfsr < 0.05), reinforcing my concern about misleading interpretations from the power-FDR plots. At these thresholds, power differences are minimal—often only in the second digit—and visible only in the supplementary tables. These results are hard to discern in the plots due to the scale. As stated in previous reviews, users need to understand how methods perform at realistic thresholds.

While the authors have acknowledged inconsistencies and corrected some plots, issues remain. In my last review, I noted inconsistencies in Supp Figs 3 and 5 and stated: "PIP/MPP thresholds in the remaining supp figures are not stated, so unclear if 0.95 is used, or if there has been an incorrect increase to 0.99 there too." This remains unresolved in Supp Figs 6, 7, and others. Given I already raised this in the previous revision, it is something the authors could have resolved.

Our response to Reviewer 3's comments

We are pleased that the reviewer now considers our paper to reach acceptable levels of transparency. We respond here to the reviewer's additional comments that refer to specific results in our paper.

Regarding "Supplementary tables show FDR is generally <0.05 at lenient thresholds (PIP > 0.80, *lfsr* < 0.05)… at these thresholds, power differences are minimal—often only in the second digit—and visible only in the supplementary tables". While these statements may be true for some comparisons, they are simply not true in general. For example, in Supplementary Table 1, look at the results for Fig. 2, Panel A at PIP > 0.8: CAFEH has an FDR of 0.23 (Scenario a) and 0.51 (Scenario b), both of which greatly exceed the (desired) FDR of 0.05. Similarly, in Supplementary Table 1, look at the results for Fig. 2, Panel B at PIP > 0.8: in Scenario a, CAFEH has a power of 0.33 compared with power or 0.44 for mvSuSiE, which is not a "minimal difference" visible only in the second digit. And in Scenario b, the power difference is even larger.

More generally, we believe our paper narrative accurately highlights the advantages of mvSuSiE over existing methods, focussing primarily on the speed and flexibility of mvSuSiE rather than gains in power. Indeed, the title of the paper is "Fast and flexible joint fine-mapping of multiple traits…", and the introduction says "Although several methods have been developed for multi-trait fine-mapping, these methods have important practical limitations. For example, several methods are computationally impractical for more than a small number of traits… Here we introduce mvSuSiE, a fast and flexible method for multi-trait fine-mapping… mvSuSiE is computationally practical for jointly fine-mapping many traits in 'biobank scale' data." Our main example highlights this last feature by jointly fine-mapping 16 traits in the UK Biobank, something most existing methods simply cannot do; for example, PAINTOR is computationally very slow even for 2 traits, and flashfm is limited to 5 traits, as highlighted in Table 1. So even though we believe the simulation studies are helpful to show that mvSuSiE is competitive with other methods in terms of power, we do not view power gains as the most important contribution here, and the paper narrative reflects this. Most importantly, the reviewer statement "it becomes clear that the new approach offers no significant gains over existing multi-trait methods" is simply untrue, ignoring large parts of our paper and results.

Regarding the comment "PIP/MPP thresholds in the remaining supp figures are not stated…" and "This remains unresolved in Supp Figs 6, 7, and others", we do not understand this comment because Supplementary Figures 6 and 7 do not involve any PIP/MPP thresholds.

These figures are about CSs, not PIPs. These figures show performance of 95% CSs (which is the only coverage level of CS considered throughout the paper), and this is indicated in the caption for Fig. 2—as well as in the captions for other figures—which says that the target coverage is 95%. Supplementary Fig. 7 also showed the target coverage as a dotted line at 0.95 but this information was not repeated in the caption for this figure; we have now added this information to the caption for Supplementary Fig. 7 (and similarly for Supplementary Fig. 10).
